# Machine Learning-Driven Design of Experiments for CO_2_ Mineralization of Steel Slag Materials

**DOI:** 10.1021/acsomega.6c07267

**Published:** 2026-10-19

**Authors:** Hamid Radfarnia, Iris Samputu, Kourosh Zanganeh

**Affiliations:** Natural Resources Canada, 113679 CanmetENERGY, 1 Haanel Drive, Ottawa, Ontario K1A 1M1, Canada

## Abstract

The mineralization of CO_2_ using steel slag represents a promising pathway for simultaneous carbon sequestration and industrial-waste valorization. However, the complex and nonlinear interactions between process variables hinder the identification of the dominant mechanisms and optimal operating conditions. In this study, a hybrid modeling framework integrating artificial neural networks (ANNs) with factorial design analysis was developed to predict and interpret the performance of three key process routes: leaching, direct carbonation, and indirect carbonation. A comprehensive data set compiled from 58 published studies, comprising 171 leaching, 168 direct carbonation, and 22 indirect carbonation experiments, was used to train and validate ANN models. The trained models were subsequently employed to generate a full factorial design (3^*k*^), enabling systematic evaluation of parameter significance and interaction effects. The ANN models demonstrated strong predictive capability for leaching and acceptable performance for carbonation processes, with improved accuracy upon inclusion of reaction time, highlighting the importance of kinetic effects. The ANN–factorial analysis suggests that leaching is most strongly associated with feed composition (CaO content) and leaching agent chemistry, while direct carbonation is controlled by reaction kinetics and surface accessibility, with strong interactions between CO_2_ availability, pretreatment, and time. Indirect-carbonation results, which are based on the smaller data set, suggest dominant associations with solution-phase chemistry, particularly pH, Ca concentration, and precipitation kinetics, with interaction effects playing a critical role in determining carbonation efficiency. Across all processes, chemical and kinetic interactions were found to be more influential than purely physical parameters. The integration of ANN with factorial design enables the creation of a virtual experimental space, allowing comprehensive exploration of complex multivariable systems beyond experimental limitations. Steel slag mineralization exhibits a progressive shift in dominant control mechanisms, from composition-dominated behavior during leaching to coupled composition–gas-supply control during direct carbonation, and finally to solution-phase control during indirect carbonation. Comparison with our previously studied Mg-rich serpentine system further shows that the ANN–factorial framework is transferable across alkaline feedstocks, while the controlling mechanisms remain feedstock-specific: Mg-rich systems are more strongly governed by pretreatment and pH/additive chemistry, whereas steel slag systems are dominated by reactive Ca availability, CO_2_ supply, and Ca–carbonate precipitation chemistry. These findings provide a screening framework for process optimization and highlight the importance of material-specific modeling in CO_2_ mineralization technologies.

## Graphical abstract

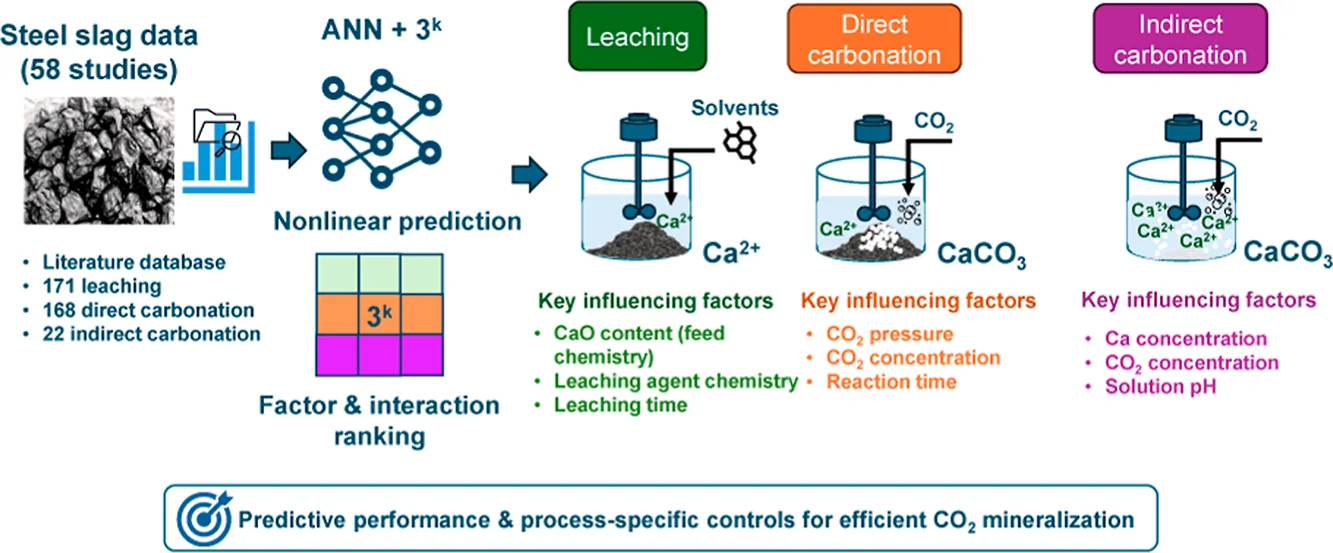

## 1 Introduction

Carbon capture and sequestration strategies have grown in popularity due to the increase in concern about greenhouse gas emissions and the resulting worldwide effort to address the CO_2_ emissions.^1,2^ Among various approaches offering CO_2_ mitigation, mineral carbonation is known as a promising technology that mimics natural weathering processes to permanently fix CO_2_ into stable carbonate minerals, offering long-term storage with minimal environmental risks.^1,3,4^ This method leverages alkaline materials rich in calcium (Ca) and magnesium (Mg), such as natural silicates like serpentine or industrial byproducts like steel slag, to react with CO_2_ and form carbonates.

Steel slags, generated in vast quantities from steelmaking processes, represent an attractive feedstock for mineral carbonation due to their high CaO content (typically 28–65 wt %), inherent alkalinity, and potential for waste valorization, thereby addressing both CO_2_ emissions and industrial waste management challenges.^5–7^

Six primary types of steel slags are commonly identified based on the production method: Ladle Furnace (LF) slag, Basic Oxygen Furnace (BOF) slag, Electric Arc Furnace (EAF) slag, Blast Furnace (BF) slag, Steel Converter slag, and Argon Oxygen Decarburization (AOD) slag.^8–10^ Each slag type has distinct chemical and mineralogical characteristics influenced by its formation process. Table 1 presents the typical chemical composition of steel slags.

**Table 1:** Typical Chemical Composition Ranges of Major Steel Slag Types^a^

component (wt %)	LF slag	BOF slag	EAF slag	BF slag	AOD slag
CaO	38–65	30–55	20–40	28–51	34–60
MgO	3–20	5–15	3–15	4–11	2–10
Al_2_O_3_	2–20	1–6	2–12	6–20	1–12
SiO_2_	12–30	10–23	6–32	26–40	24–32
Fe_2_O_3_	0.7–7	10–37	20–40	<2	0.5–12
TiO_2_	<1	0.5–2	<2	<2	<1
MnO	0.4–4	0.7–5	0.4–7	<1	<1
Cr_2_O_3_	<1	<1	1–6	<1	<3
P_2_O_5_		1–3	0.5–2		
pH	11–13	11–13	10–12	10–11	12–13

a Data compiled and adapted from refs 7 and 10 . Adapted in part from DiGiovanni, C.; Hisseine, O. A.; Awolayo, A. N. *J. CO*_*2*_ *Util.* **2024**, DOI: 10.1016/j.jcou.2024.102736. Copyright 2024 Published by Elsevier Ltd. Adapted in part from Biava, G.; Depero, L. E.; Bontempi, E. *Span. J. Soil Sci.* **2024**, *14*, 12908. Copyright 2024 Biava, Depero, and Bontempi. Distributed under the Creative Commons Attribution License (CC-BY).

The composition of steel slag varies depending on the specific production process and the materials introduced during manufacturing. However, it generally contains substantial amounts of calcium (Ca) and magnesium (Mg) in various forms, such as oxides, silicates, aluminosilicates, and the RO phase, an inert solid solution of CaO–FeO–MnO–MgO. Additionally, steel slag retains trace elements from the steelmaking process, including Fe, Mn, Cr, Ti, S, and V. Reported mineralogical analyses indicate that steel slags typically contain approximately 15%–25% dicalcium silicate (Ca_2_SiO_4_, C_2_S), 20%–25% tricalcium silicate (Ca_3_SiO_5_, C_3_S), and 40%–45% RO phases (an inert solid solution of CaO, FeO, MnO, and MgO) along with minor amounts of free CaO and MgO, although these values vary with slag type and cooling history.^7,10,11^ DiGiovanni et al.^7^ noted that due to the typically slow cooling rate of steel slag and its high basicity ratio (CaO/SiO_2_), the formation of tricalcium silicate and β-dicalcium silicate is limited.

Since steel slag is a byproduct of steel manufacturing, its production is directly proportional to crude steel output. The World Steel Association reported that global crude steel production in 2024 reached approximately 1.885 billion tons.^12^ This upward trend in global steel production capacity reinforces the abundant availability of steel slag, positioning it as a promising candidate for CO_2_ sequestration applications. According to the U.S. Geological Survey (USGS),^13^ steel slag production globally was estimated to be in the range of 190–290 million tons in 2023, approximately to be 10% to 15% of raw steel production. To accommodate this increase in byproduct waste materials, more landfills and associated infrastructure are needed, leading to additional cost for plant operators, as well as for managing their adverse environmental impact and waste disposal.

Mineral carbonation of steel slags can proceed via direct or indirect routes, each influenced by key operational parameters that govern reaction efficiency, CO_2_ uptake, and product characteristics.^7,14–16^ In the direct carbonation method, steel slag is reacted with CO_2_ in a single step, typically under aqueous or gaseous conditions, to form carbonates directly within the slag matrix.^17,18^ This approach is simpler and more energy-efficient but often limited by CO_2_ mass transfer, surface passivation by precipitated carbonates, and slow dissolution kinetics of alkaline phases.^18,19^ Critical operational parameters include temperature (optimally 50–150 °C to enhance kinetics without decomposing carbonates), CO_2_ pressure (1–20 bar to increase solubility and reaction rate), particle size (finer particles <100 μm increase surface area and reactivity), liquid-to-solid ratio (0.5–10 L/kg to facilitate ion diffusion), and reaction time (0.5–24 h to achieve equilibrium).^7,20^

These parameters are vital as they directly impact the carbonation rate, where higher temperatures and pressures accelerate dissolution and precipitation but may raise energy costs, while optimal particle size minimizes diffusion barriers.^21,22^ The indirect carbonation route, in contrast, involves a two-step process: initial leaching of Ca^2+^ and/or Mg^2+^ ions from the slag using acids or solvents, followed by carbonation of the extracted solution with CO_2_ to precipitate pure carbonates. This method achieves higher purity products and greater CO_2_ sequestration potential (up to 70–90% conversion) but requires additional chemical inputs and separation steps, making parameter optimization essential for economic viability.^23,24^

For the leaching step, key parameters encompass acid type and concentration (e.g., 0.5–2 M acetic or hydrochloric acid for selective ion extraction), temperature (25–80 °C to boost solubility without excessive energy use), stirring speed (200–800 rpm for improved mass transfer), acid-to-slag ratio (1–5 mol/kg to balance extraction yield and acid consumption), and leaching time (0.5–4 h to maximize ion yield while minimizing heavy metal coleaching).^5,7,25,26^ These factors are crucial as they determine leaching efficiency, pH-dependent ion selectivity, and the environmental safety of residues, with improper settings leading to incomplete extraction or unwanted contaminants.

In the subsequent carbonation phase, parameters often mirror those of direct methods but emphasize pH control (8–12 for optimal carbonate precipitation), CO_2_ flow rate (to maintain saturation), and agitation to prevent agglomeration.^27,28^

The importance of these parameters lies in their collective influence on overall process kinetics, energy consumption, and scalability; for instance, elevated temperatures enhance reaction rates but may reduce the level of CO_2_ solubility, necessitating a balanced factorial design for optimization.

Despite recent advancements in steel slag mineralization for CO_2_ sequestration, several critical gaps and challenges persist that hinder its widespread adoption. Key limitations include low carbonation efficiency and slow reaction kinetics, primarily due to the formation of dense carbonate product layers that impede CO_2_ diffusion and mass transfer, resulting in incomplete conversions and unreacted cores within slag particles.^16,26,28^ The heterogeneous and variable chemical composition of steel slags, influenced by steelmaking processes and cooling methods, leads to inconsistent reactivity and challenges in standardizing treatment protocols.^7,10^ Additionally, high energy demands for preprocessing steps like mechanical grinding to achieve optimal particle sizes (<150 μm) and heat treatments, coupled with the need for chemical additives in indirect methods, raise economic and environmental concerns, including increased costs and potential leaching of heavy metals such as Cr and V.^7,10^ Scalability remains a major hurdle, with most research limited to laboratory or pilot scales, lacking comprehensive life cycle assessments, long-term durability studies of carbonated products, and evaluations of industrial viability.^7,10^

These gaps underscore the need for advanced predictive tools to optimize process parameters, enhance reaction kinetics, and facilitate scale-up, thereby improving the overall efficiency and sustainability of steel slag-based CO_2_ sequestration.

Our previous research has demonstrated the value of artificial neural networks (ANNs) integrated with 3^*k*^ factorial experimental design in modeling and predicting the behavior of leaching and carbonation processes for serpentine-based systems.^29^ This approach enabled accurate optimization of process variables, yielding insights into reaction and efficiency for CO_2_ sequestration via indirect and direct carbonation. Most studies on steel slag mineralization employ a one-factor-at-a-time experimental approach, typically focusing on calcium carbonation from a single feedstock source. This limits the exploration of complex interactions among variables and may hinder comprehensive process optimization due to the lack of time and materials for comprehensive factor analyses. To address these resource limitations efficiently, the adaptability and predictive capabilities of ANN offer a promising solution for modeling and optimizing steel slag CO_2_ mineralization processes.

Machine learning effectively addresses nonlinear, multivariable systems, with ANNs particularly outperforming traditional models due to their ability to handle complex, nonlinear systems, and high-dimensional data,^30,31^ in particular in the context of CCUS.^32,33^

Building upon this framework, the present study expands the methodology to cover steel slags, an abundant and highly reactive material with significant potential for CO_2_ sequestration, while simultaneously addressing the environmental challenge of a hazardous waste that would otherwise contribute to landfill accumulation and heavy metal leaching. Although a few studies have applied factorial design in the context of steel slag’s CO_2_ mineralization,^34–36^ they predominantly focus on experimental investigations within narrowly defined boundary conditions.

To the best of our knowledge, no previous study has integrated machine learning-based predictive modeling, leveraging existing experimental data, with a full factorial design to analyze the influence of key parameters on the carbonation of steel slag materials. In this study, an ANN is combined with a 3^*k*^ factorial design to enhance predictive modeling for scalable CO_2_ mineralization using steel slags, while systematically examining the synergistic effects of interacting operational parameters on system performance. This approach offers critical insights into the predicted impact of various combinations of process factors, which are typically impractical to investigate experimentally. To support this, a comprehensive experimental data library was first compiled from the literature. Because the database combines experiments from different studies, slag sources, analytical methods, and reactor configurations, the resulting model should be interpreted as a literature-domain screening tool rather than as a universal predictor. The diversity of the data set broadens the operating space captured by the ANN, but it may also introduce study-level heterogeneity and latent confounding. These limitations are addressed through grouped validation, bootstrap analysis, and cautious interpretation of ANN-derived factor rankings.

## 2 Methodology for Multivariate Analysis

The methodology used for the integrated ANN–factorial framework in this study is presented in Figure 1. Briefly, literature-derived data sets are used to train ANN models for nonlinear predictive modeling of CO_2_ mineralization processes. The trained models are then coupled with a full factorial design (3^*k*^) to generate a virtual data set, enabling systematic sensitivity and interaction analysis. This combined approach allows both prediction of process performance and model-assisted interpretation of dominant parameters and their interactions.

**Figure 1:**
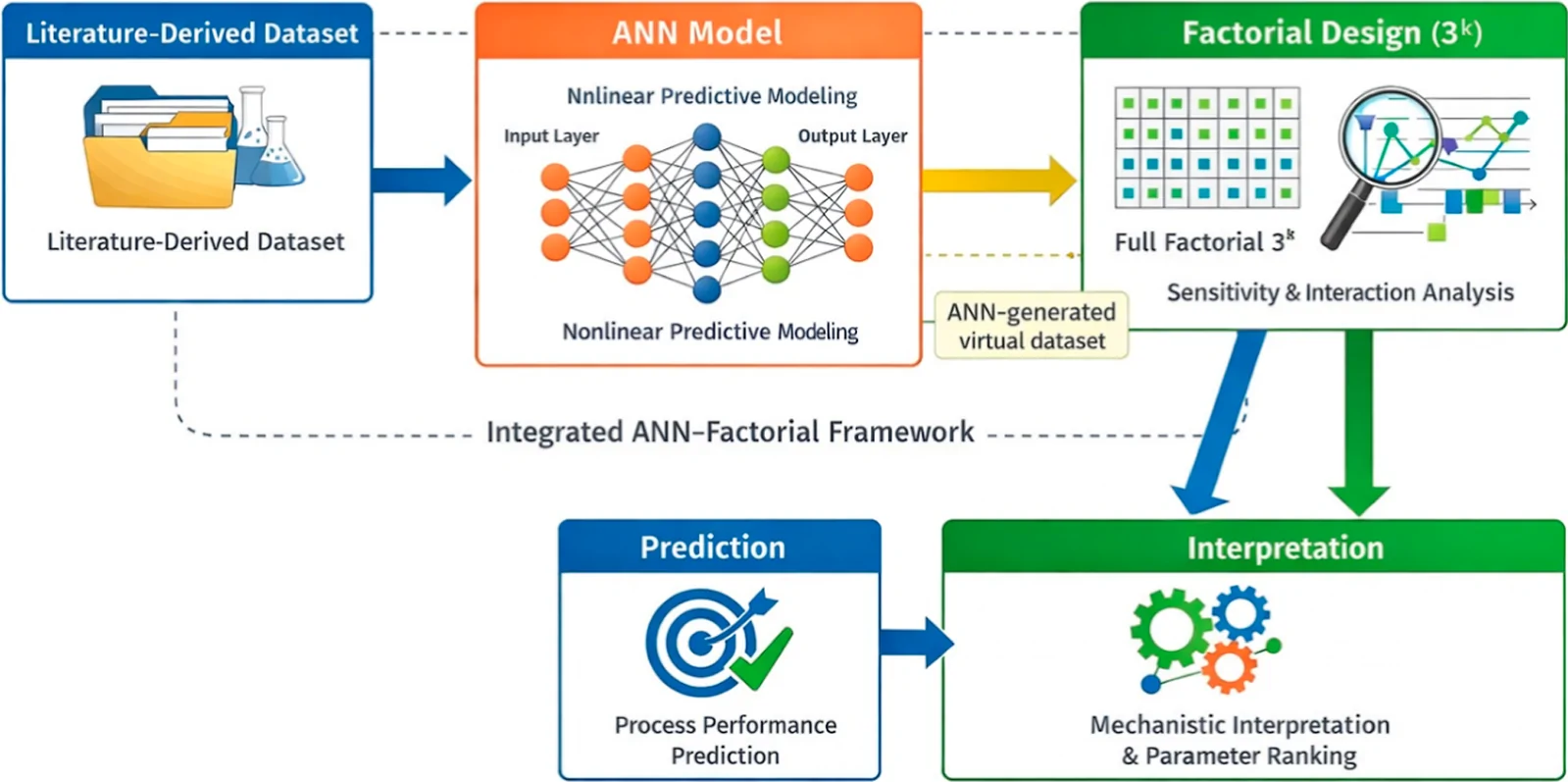
Schematic representation of the integrated ANN–factorial framework used in this study.

The practical role of the ANN model in this study extends beyond fitting the historical literature data. Within the composition and operating domain represented by the compiled database, the trained ANN can be used as a surrogate screening tool to estimate likely process responses for steel slags with known input characteristics. Such predictions are intended to support the preliminary evaluation and prioritization of operating conditions rather than to replace source-specific experimental validation. This distinction is particularly important for previously unseen slag sources, which may differ in mineralogy, free-CaO content, cooling history, Fe-bearing phases, and reactive Ca availability, even when their bulk chemical compositions are similar.

Similar hybrid ANN-plus-DOE strategies have been used in multivariable process systems specifically because ANN provides predictive power while factorial analysis restores interpretability and reduces the number of experiments needed for optimization.^37–39^

### 2.1 Literature Search and Screening

A reproducible literature search and screening protocol was used to construct the modeling database. Searches were conducted in Scopus and Google Scholar last dated on October 15, 2025 using combinations of the following search strings: steel slag, CO_2_ mineralization, CO_2_ sequestration, mineral carbonation, calcium carbonate, EAF slag, BF slag, BOF slag, aqueous mineralization, AOD slag, LF slag, ladle metallurgical furnace slag, and slurry carbonation. The search targeted studies related to steel slag, Ca-bearing industrial residues, leaching, direct carbonation, indirect carbonation, CO_2_ mineralization, mineral carbonation, and CO_2_ sequestration.

Duplicate records were removed by comparing the title, DOI, author list, and publication year. Studies were retained when they reported quantitative experimental or process data for Ca-bearing steel slags relevant to leaching, direct carbonation, or indirect carbonation. Studies were excluded when they did not report extractable numerical data, used nonslag feedstocks only, duplicated previously reported data sets, lacked sufficient process-variable information, or provided only qualitative review discussion.

The initial bibliometric search yielded 1097 records for the keyword-mapping analysis. After keyword filtering and topic screening, approximately 252 articles were retained for a detailed consideration. Further screening identified 74 experimental steel-slag studies, of which 58 contained sufficient quantitative information for the ANN model development. The final data set contained 171 leaching observations, 168 direct-carbonation observations, and 22 indirect-carbonation observations. A PRISMA-style screening diagram and the final article library are provided in the Supporting Information, Figure S1.

To visualize topical trends and keyword relationships, a bibliometric map was generated by using VOSviewer software (Figure 2). The map groups frequently co-occurring keywords into thematic clusters and illustrates publication and citation relationships within the steel slag CO_2_ mineralization literature. The colored clusters represent interconnected research themes, including CO_2_ sequestration and leaching, carbonate product formation, and steel-slag-related mineralization studies.

**Figure 2:**
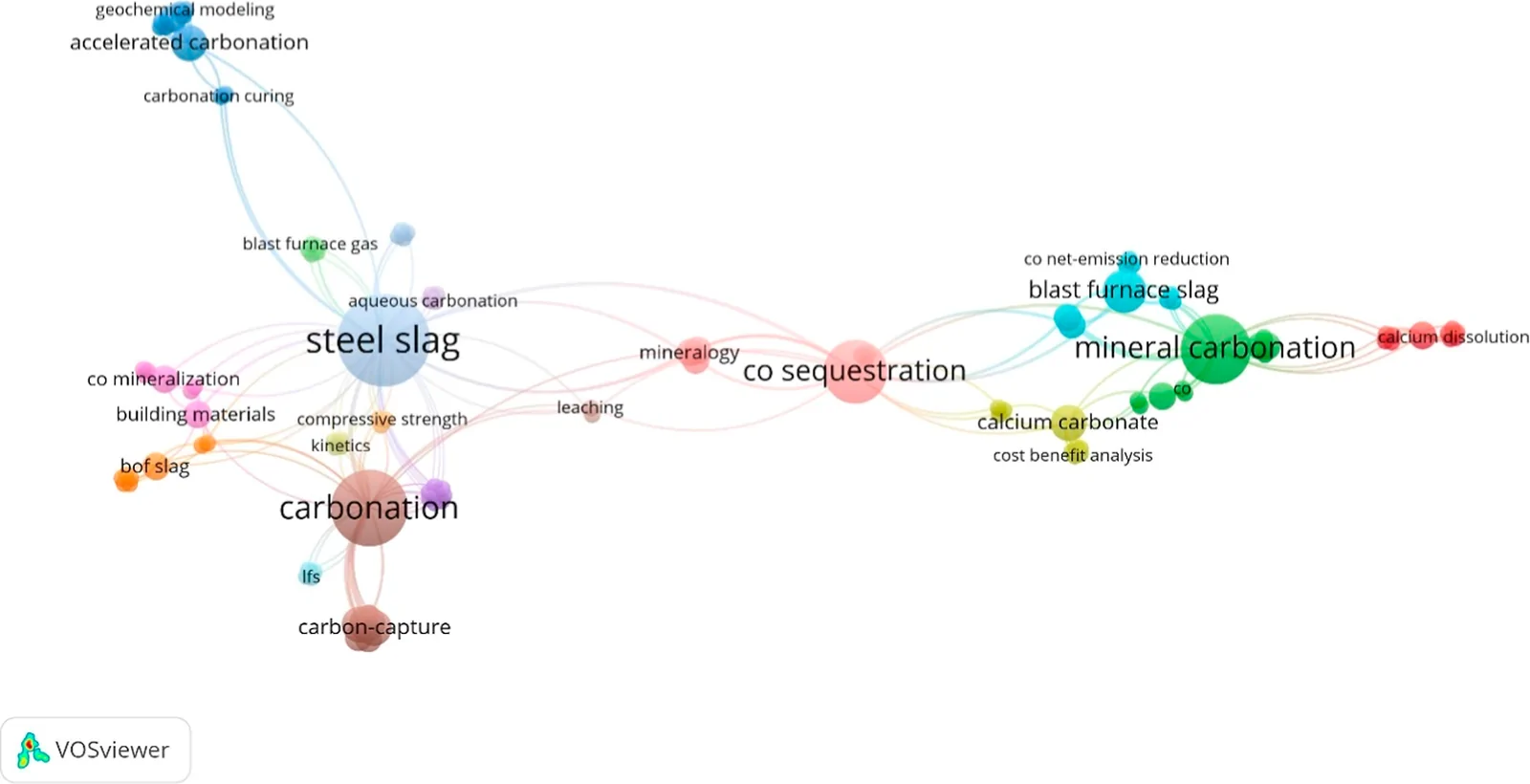
Bibliometric map of keywords created with VOS software data created from 1097 articles from 1998 to 2025. The circle sizes correspond to the frequency of keyword occurrences, and related research topics are organized into distinct colored clusters.

Among the 58 studies included in the detailed database, 10% were published before or in 2010, 28% from 2011 to 2015, 24% from 2016 to 2020, and 38% after 2020. Approximately 8% focused on blast-furnace slag, 20% on electric-arc-furnace slag, 38% on basic-oxygen-furnace slag, 12% on ladle-furnace slag, and 3% on argon–oxygen-decarburization slag, with the remainder involving unspecified steel slags. The distribution of studies by process route and the individual articles used for model development are summarized in Table S1.

### 2.2 ANN Architecture and Validation

ANN models were developed separately for leaching, direct carbonation, and indirect carbonation. Multiple ANN architectures were evaluated by varying the network depth from 10 to 100 hidden layers (10, 20, 50, and 100 layers) to identify the architecture that provided the best balance between predictive accuracy and generalization. Because these architectures are relatively high capacity compared with the available data set sizes, model selection was based not only on training performance but also on validation and testing performance combined with early stopping behavior. The complete architecture of the final model for each process route is summarized in Table S8.

A comparative evaluation of several training algorithms was conducted, including Levenberg–Marquardt, BFGS quasi-Newton, scaled conjugate gradient, Fletcher–Powell conjugate gradient, variable learning-rate backpropagation, Polak–Ribiere conjugate gradient, and one-step secant. Among these, the Levenberg–Marquardt algorithm provided the best overall performance and was selected for the final ANN models. Model performance was evaluated using correlation coefficient (*R*), root mean squared error (RMSE), MSE, and mean absolute error.

For leaching and direct carbonation, the observation-level data sets were initially partitioned into 80% training, 10% validation, and 10% testing subsets. For indirect carbonation, the smaller data set was partitioned into 50% training, 25% validation, and 25% testing subsets. A complementary grouped-validation analysis, in which observations originating from the same publication or slag source are retained within the same fold, is planned to further test generalization to unseen studies or sources; results will be reported separately.

Model stability was further assessed by an input-perturbation sensitivity analysis and bootstrap resampling (*n* = 500). For the bootstrap analysis, ANN models were independently retrained for each resampled data set using the same model-development procedure. Confidence intervals and rank-stability estimates derived from this analysis will be reported separately in a methodology-focused manuscript. A detailed description of the general ANN-development framework is also available in Samputu et al.^29^

After model development and validation, the trained ANN models were used to predict extraction efficiency and CO_2_ sequestration capacity across three full factorial virtual experimental designs: 3^7^ combinations for leaching, 3^8^ combinations for direct carbonation, and 3^6^ combinations for indirect carbonation, corresponding to 2187, 6561, and 729 virtual experiments, respectively. These ANN-generated response surfaces were subsequently used for a surrogate-based factorial sensitivity and interaction analysis.

### 2.3 3^*k*^ Factorial Design and Parametric Statistical Analysis

To investigate the individual and interactive effects of process parameters on steel slag mineralization, a 3^*k*^ factorial design was employed. This approach evaluates three levels—low, medium, and high—for each factor and therefore provides greater resolution than traditional 2^*k*^ designs, which consider only minimum and maximum levels.^40,41^ The 3^*k*^ design is particularly useful when intermediate values may influence process responses, as is often the case in leaching and carbonation systems.^29,36,42–46^ For the steel slag data sets considered here, the factorial design included seven factors for leaching, eight factors for direct carbonation, and six factors for indirect carbonation, resulting in 3^7^, 3^8^, and 3^6^ factorial combinations, respectively. The corresponding factors and levels are summarized in Tables 2–4.

**Table 2:** 3^*k*^ Factorial Design Parameters for the 2187 Leaching Experiments

	values					
	low (0)		medium (1)		high (2)	
parameter studied	100% data	80% data	100% data	80% data	100% data	80% data
Factor 1-CaO feed content (%)	32.3	38	46.5	42.6	60.7	47.2
Factor 2-particle diameter (μm)	3000	1000	1519	522.5	38	45
Factor 3-solid/liquid ratio (g/L)	200	100	102	55	4	10
Factor 4-reaction temperature (°C)	15	22	52.5	43.5	90	65
Factor 5-time duration (h)	0.08	0.2	1.29	1.1	2.5	2
Factor 6-auxiliary treatment	no treatment		mild activation (hypothetical)^a^		microwave/ultrasound treatment	
Factor 7-leaching agent	other reagents (including water and CO_2_ solution)		organic acids		inorganic acids	

a Auxiliary treatment (medium) represents a hypothetical intermediate level introduced for numerical modeling purposes and may be interpreted as moderate activation such as mechanical activation or mild heating.

**3:** 3^*k*^ Factorial Design Parameters for the 6561 Carbonation Experiments—Direct^a^

	values					
	low (0)		medium (1)		high (2)	
influencing factors	100% data	80% data	100% data	80% data	100% data	80% data
Factor 1-CaO feed content (%)	20.9	31	39.5	40.5	58.1	50.1
Factor 2-particle size (μm)	2000	200	1005	122	10	44
Factor 3-solid/liquid ratio (g/L)	50	67	5025	1283.5	10,000	2500
Factor 4-reaction temperature (°C)	20	20	97.5	60	175	100
Factor 5-CO_2_ pressure (MPa)	0.005	0.015	6.0	4.2	12	8.3
Factor 6-CO_2_ concentration (%)	5	15	52.5	57.5	100	100
Factor 7-carbonation assisting agent	none used		MEA		NaHCO_3_, K_2_CO_3_	
Factor 8-time (h)	0.16	0.67	24.1	24.3	48	48

a Solution pH was not included as an independent factor because the majority of direct carbonation studies relied on the intrinsic alkalinity of the slag system, resulting in insufficient variability for a meaningful factorial analysis.

**Table 4:** 3^*k*^ Factorial Design Splits for the 729 Carbonation Experiments—Indirect^a^

	values					
	low (0)		medium (1)		high (2)	
influencing factors	100% data	80% data	100% data	80% data	100% data	80% data
Factor 1-Ca extracted (M)^b^	0.004	0.017	0.36	0.17	0.72	0.32
Factor 2-reaction temperature (°C)	22	25	51	45	80	65
Factor 3-reaction initial pH	5.4	7.2	8.7	8.4	12	9.5
Factor 4-CO_2_ pressure (MPa)	0.01	0.013	0.51	0.056	1	0.1
Factor 5-CO_2_ concentration (0–100%)	10	12.5	55	56.3	100	100
Factor 6-time (h)	0.12	0.26	1.06	0.63	2	1

a Insufficient data were available to support ANN training and validation for carbonation assistant agents; therefore, this factor was not included in the analysis.

b The Ca concentration in the carbonation leachate was estimated from the corresponding extraction-step test data.

The minimum, intermediate, and maximum levels were derived from the curated literature database. To assess the sensitivity of the factorial rankings to extreme reported values, two ranges were evaluated. The first used the full range of the compiled data, whereas the second retained the central 80% of observations between the 10th and 90th percentiles. This percentile-based analysis was used to reduce the influence of extreme literature values and to determine whether the principal factor rankings remained stable within the more densely represented operating range. This approach complements conventional methods for identifying and evaluating outlying observations.^47–49^

Categorical variables were initially represented using three-level numerical coding to enable their inclusion in the 3^*k*^ factorial framework. For auxiliary treatment, untreated feedstocks were assigned index 0, thermal or intermediate treatment index 1, and microwave/ultrasound treatment index 2. Leaching agents were categorized as other reagents, including water and CO_2_-containing solutions (index 0); organic acids, including CH_3_COOH (index 1); and stronger inorganic mineral acids, such as HCl and HNO_3_ (index 2). For direct carbonation, the assisting-agent factor was represented as no additive (index 0), monoethanolamine (MEA; index 1), and NaHCO_3_/K_2_CO_3_-based additives (index 2). Because such ordinal coding can impose an assumed ordering on chemically distinct categories, a sensitivity analysis using a nonordinal categorical representation is planned to test the robustness of the principal conclusions separately.

The CO_2_ concentration in the gas phase was included as an independent factor for direct carbonation. The concentration ranges were derived from the compiled literature, which predominantly employed simulated CO_2_/inert gas mixtures. Consequently, these levels represent the experimental domain available in the database and may not capture the full compositional complexity of the industrial flue gases.

For indirect carbonation, a carbonation-assisting-agent factor was not included because the collected studies did not provide sufficient variation in assisting-agent identity for independent ANN training and factorial analysis; NaOH was the represented alkaline reactant in the available data set. Consequently, the indirect carbonation factorial design contained only six factors.

The trained ANN models were subsequently used to predict the extraction efficiency or CO_2_ sequestration capacity for all virtual combinations generated by the factorial designs. This produced 2187 ANN-predicted responses for leaching, 6561 for direct carbonation, and 729 for indirect carbonation. These virtual data sets represent ANN-generated response surfaces rather than independent experimental measurements. Therefore, analysis of variance (ANOVA)^42,50^ was used here as a surrogate-response decomposition and factor-ranking method rather than as a classical statistical inference from independent experimental replicates.^51,52^

*F*-Values were calculated from the corresponding sums of squares and mean squares and were normalized within each ANN-generated response surface to compare the relative contributions of the individual factors and their interactions. Nominal *P*-values are retained as descriptive screening quantities for the surrogate model decomposition and are not interpreted as evidence of independent experimental statistical significance. To account for uncertainty associated with the ANN predictions, the ANN–DOE–ANOVA workflow was evaluated across 500 bootstrap-trained ANN models. Although uncertainty associated with the ANN predictions was assessed through bootstrap analysis, the resulting confidence intervals and rank stability estimates are not discussed here as uncertainty quantification was not a primary focus of the present study. The ANN–DOE–ANOVA methodology, including the simplified ANOVA model specification and residual structure, is described in Supporting Note S1 of the Supporting Information.

The indirect-carbonation data set contained only 22 observations. Consequently, the corresponding ANN predictions and factorial rankings should be interpreted as exploratory indicators of dominant trends, rather than definitive mechanistic rankings. These results are retained because they provide preliminary insight into the relative importance of solution-phase variables, although their ranking uncertainty is greater than that associated with the leaching and direct-carbonation data sets.

## 3 Results and Discussion

### 3.1 Extraction Analysis

#### 3.1.1 ANN Modeling with Literature Data

ANN modeling was employed to develop and train the predictive model for such nonlinear and complex systems, where multiple interacting parameters simultaneously influence the process performance. The model’s effectiveness was evaluated using the coefficient of determination (*R*) and MSE. The experimental data set was split into 80% for training, 10% for validation, and 10% for testing purposes. The final model used for data extraction yielded an average MSE of 16.0456 and an *R* value of 0.9816 across all data subsets. Additionally, the percentage RMSE was approximately ±4.0%, indicating a strong agreement between the model predictions and the experimental observations. A graphical representation of the model’s performance is provided in Figure 3.

**Figure 3:**
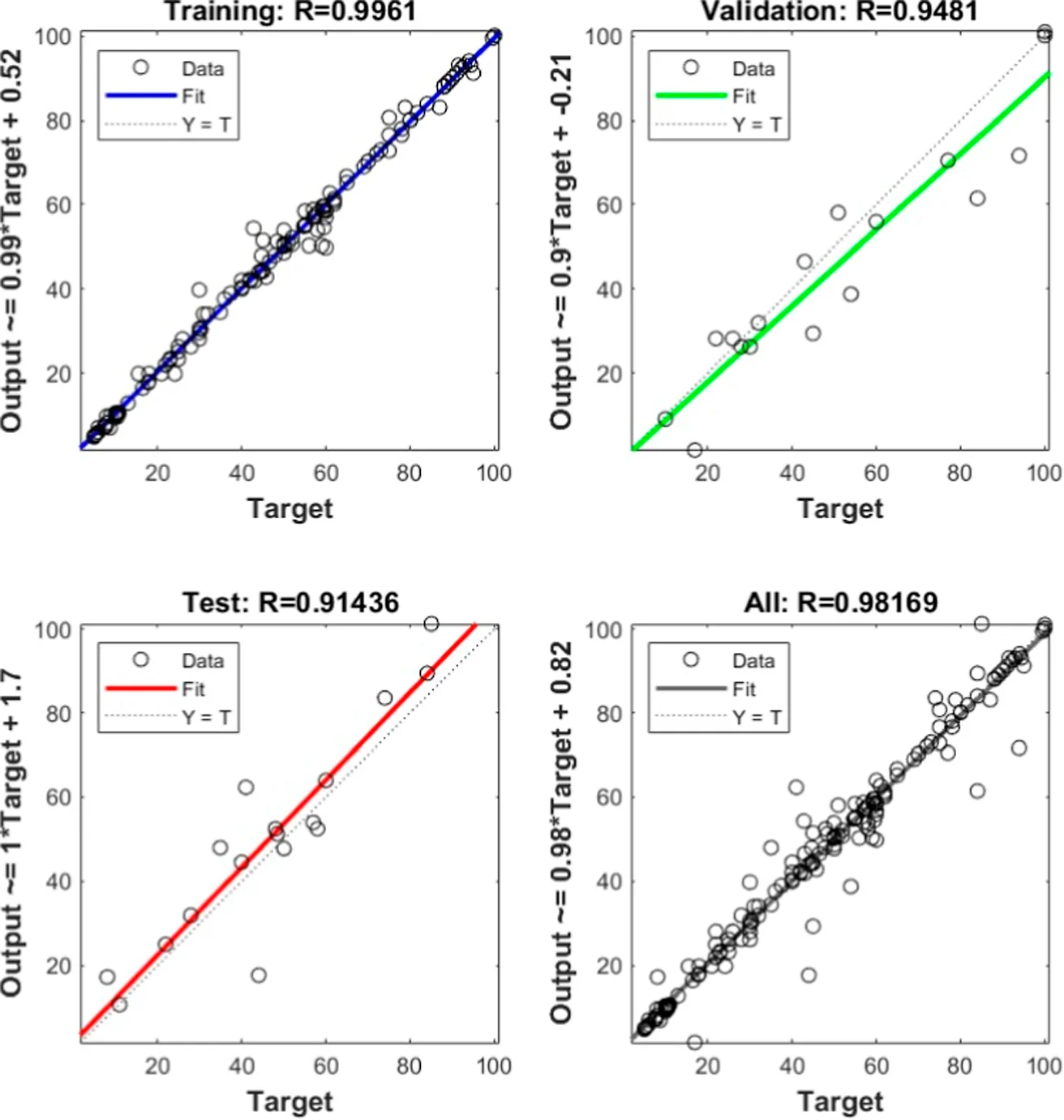
Extraction efficiency ANN analysis results for steel slag data.

To evaluate the ANN model’s sensitivity to input variations, a perturbation analysis was performed by incrementally altering each factor (CaO content, particle size, solid/liquid ratio, temperature, time duration, additional treatment, and leaching agent) from 0.1% to 50% of their baseline values, with other inputs fixed. The resulting changes in root-mean-square error (RMSE) quantify prediction instability, as shown in Figure 4. Steeper or more volatile RMSE curves denote higher sensitivity, signaling factors where small changes disproportionately affect leaching efficiency forecasts. CaO content displayed the most pronounced fluctuation (orange line), with RMSE rising sharply from near 0% at 0.1% perturbation to ∼80% error by ∼25% perturbation, then dropping to ∼70% at 35%–40% perturbation, before surging again to over 90% error at 50%. The nonmonotonic RMSE response may reflect several factors, including heterogeneous phase distributions within the underlying slag data, variations in the representation of Ca-rich slags within the training data set, or limitations in model extrapolation beyond the dominant composition range. Consequently, the observed behavior should be interpreted as evidence of strong model sensitivity to CaO content rather than as confirmation of a specific physical mechanism. The leaching agent ranked second in overall sensitivity (brown line), with RMSE climbing steadily to near 70% at 50% perturbation. As a core chemical driver, even modest changes (e.g., change in acid concentration) disrupt pH gradients and ion selectivity, increasing errors as the model’s reliance on protonation capability of solvent used which directly influences the Ca^2+^ dissolution rate. Time duration was placed third (light green line), reaching ∼40% RMSE at 50% perturbation. This moderate rise implies the model robustness in short-term regimes, where extensions yield diminishing returns without major forecast disruptions. Additional treatment (dark blue line) and temperature (purple line) followed with similar profiles, both peaking at ∼30% RMSE. Treatments (e.g., ultrasonication intensity) introduce auxiliary mechanical effects with limited standalone volatility, while temperature perturbations in the thermal window of literature data (up to 90 °C) will not be significant and keeping errors contained unless exceeding optimal ranges that risk gel formation or reduced solubility. Physical factors showed the least impact: solid/liquid ratio (cyan line) and particle size (dark green line) stayed below 10% RMSE across perturbations. These reflect stable mass transfer roles, where gradual shifts in S/L ratio caused minor dilution without cascading effects, and particle size changes align with surface area scaling already embedded in the model, confirming their lower-order influence in ANN predictions. In summary, chemical factors (CaO, leaching agent) accounted for the most sensitivity variance through volatile, threshold-driven responses, while physical ones (S/L, particle size) contributed much less.

**Figure 4:**
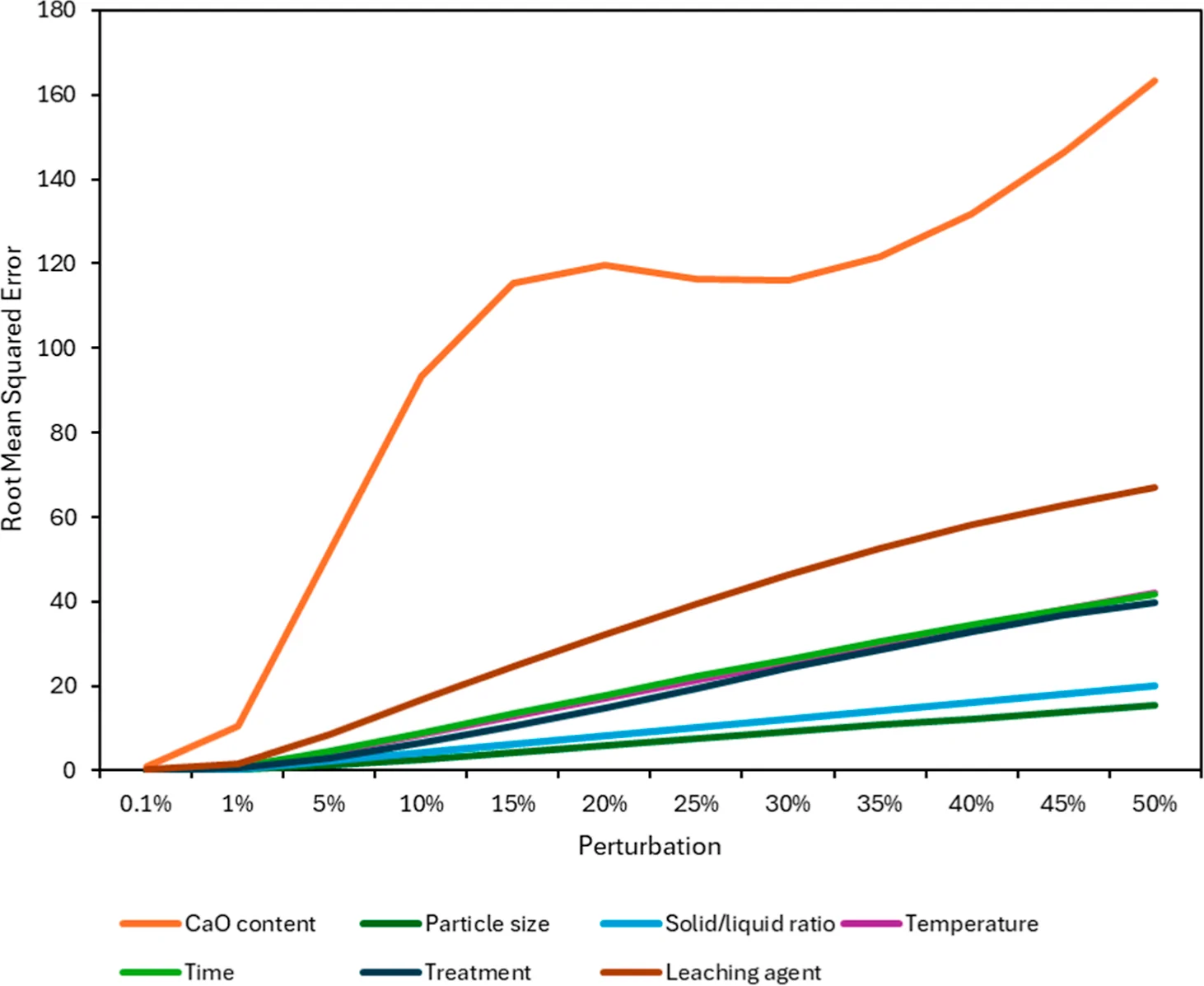
Extraction ANN sensitivity analysis results for steel slags.

#### 3.1.2 3^*k*^ Factorial and ANOVA Results

A full factorial 3^7^ experimental design was used to evaluate the effects of seven factors on the leaching efficiency from steel slags for CO_2_ mineralization. Low, medium, and high levels for each factor were set on the basis of literature data. Two data sets were analyzed: one using the full 100% range of reported values and another using a reduced 80% range to better reflect real-world variations in industrial materials. Each data set included 2187 virtual experiments. ANOVA was applied to calculate *F* values, which show the strength of each main effect and interaction. *F*-values were normalized to the highest value in each data set for easy comparison (see Supporting Information, Tables S2 and S3 for the raw *F*- and *P*-values). Results are shown in Figures 5a,b and 6a,b, as bar charts of normalized *F*-values.

**Figure 5:**
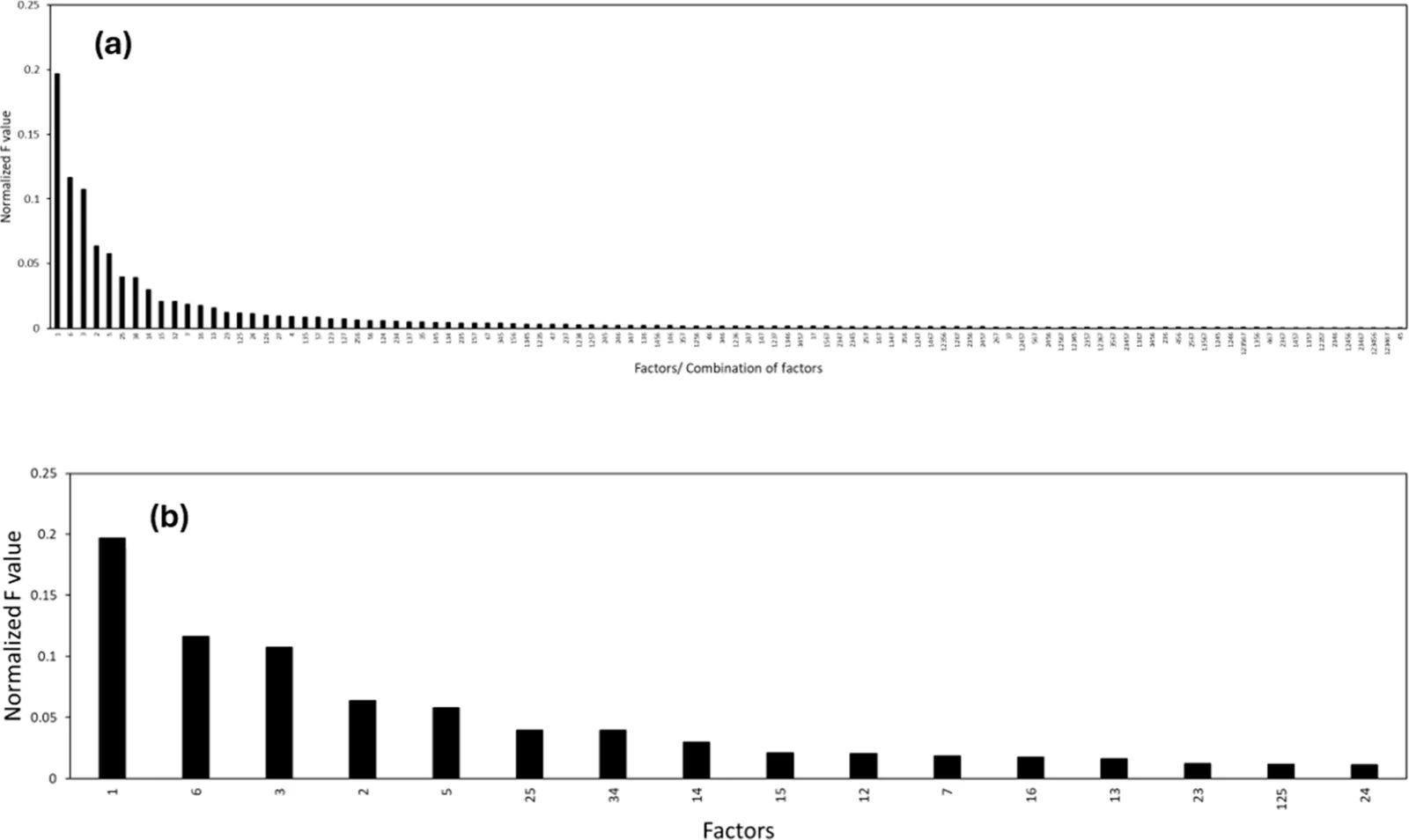
Extraction ANOVA analysis results for steel slags using 100% of data; (a) full ranking, (b) first 15 rankings.

**Figure 6:**
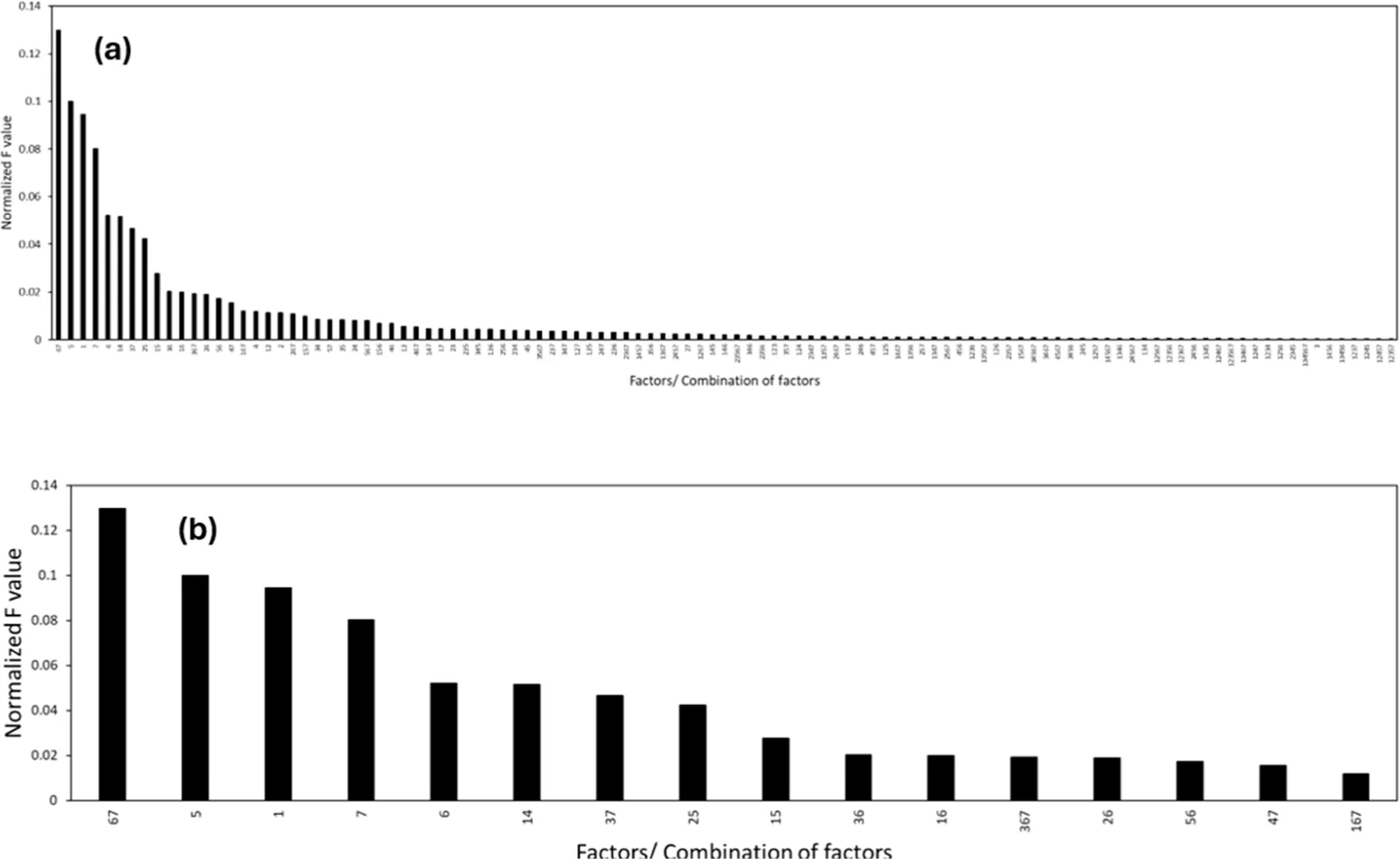
Extraction ANOVA analysis results for steel slags using 80% of data; (a) full ranking, (b) first 15 rankings.

For the 100% data set (Figure 5), the first 15 ranked terms are 1, 6, 3, 2, 5, 25, 34, 14, 15, 12, 7, 16, 13, 23, 125. The first five positions show that the main effects are still dominant when the full literature spread is preserved: CaO feed content (1) ranks first, followed by auxiliary treatment (6), solid/liquid ratio (3), particle size (2), and time (5). This means the extraction response is set first by the intrinsic reactivity of the slag, then by the extent to which pretreatment and liquid contact make that calcium accessible. High-CaO slags generally contain more free lime and reactive Ca–silicate phases, but the extent to which that potential is realized depends on whether treatment and liquid-phase conditions expose those phases effectively. The appearance of interacting factors 25 (particle size × time) and 34 (solid/liquid ratio × temperature) in the next ranks shows that kinetics and transport become important once reactive calcium is present: smaller particles need enough time to translate surface accessibility into dissolved Ca, while the solid/liquid ratio and temperature together determine both dissolution rate and saturation constraints. The next group, 14 (CaO × temperature), 15 (CaO × time), 12 (CaO × particle size), 7 (leaching agent), 16 (CaO × treatment), 13 (CaO × solid/liquid ratio), 23 (particle size × solid/liquid ratio), and 125 (CaO × particle size × time), is consistent with extraction is not driven by chemistry alone or transport alone, but by a layered hierarchy in which feed chemistry is primary and transport/activation variables determine how much of that chemistry becomes accessible. This interpretation is consistent with steel slag extraction studies showing that Ca release depends strongly on phase assemblage, solvent strength, and pH control, while particle size, contact time, and slurry conditions modulate dissolution extent rather than replacing feed chemistry as the governing factor.^5,7,53^

For the 80% data set (Figure 6), the first 15 ranked terms become 67, 5, 1, 7, 6, 14, 37, 25, 15, 36, 16, 367, 26, 56, 47. This ranking provides additional insight because the reduced data set suppresses extreme literature points and exposes the more reproducible operating logic. The strongest term, 67 (auxiliary treatment × leaching agent), shows that within the central literature-derived operating range, the process is governed less by any single variable and more by the synergy between physical activation and chemical extraction. In other words, a stronger lixiviant is most valuable when treatment has already increased exposure of reactive Ca-bearing phases, and treatment is most useful when paired with a solvent capable of exploiting that new accessibility. The next four terms, 5 (time), 1 (CaO content), 7 (leaching agent), and 6 (treatment), show that once the process window is narrowed, leaching becomes a controlled combination of residence time, slag chemistry, solvent strength, and activation history. The next ranks are also chemically meaningful: 14 (CaO × temperature) suggests that warmer conditions preferentially benefit Ca-richer slags because dissolution is accelerated only when sufficient reactive Ca is available; 37 (solid/liquid ratio × leaching agent) shows that solvent effectiveness is partly governed by dilution and acid availability; 25 (particle size × time) reflects the kinetic benefit of smaller particles only when sufficient contact time exists; 15 (CaO × time) indicates that richer slags need enough residence time to translate potential Ca inventory into dissolved Ca; 36 (solid/liquid ratio × treatment) and 16 (CaO × treatment) show that treatment benefits depend on both slurry conditions and feed composition; 367 indicates a three-way synergy where activation, solvent chemistry, and slurry loading operate as a coupled extraction environment; 26, 56, and 47 then confirm that particle size–treatment, time–treatment, and temperature–leaching-agent effects remain important but secondary. Overall, Figure 6 supports a stronger conclusion than Figure 5: within practical ranges, steel slag extraction is governed by coupled activation–chemistry effects rather than by purely physical variables.^5,53^ The different contribution of interaction terms in the 80% data sets does not indicate stronger or weaker underlying physical interactions. Removing extreme observations reduces the variance associated with dominant main effects, allowing synergistic relationships among variables to become more apparent within the typical operating range. Consequently, the 80% data sets characterize the central operating range represented by the compiled literature, whereas the full data sets capture the broader variability reported in the literature.

Extraction surface plots for steel slags using 80% of data are given in the Supporting Information (Figure S2). Figure S2A (time vs CaO content), suggesting that the influence of time becomes more pronounced at higher CaO feed contents, suggesting that time functions as a conditional enhancer of feed reactivity rather than a universally dominant factor. Where the slag contains limited reactive Ca, longer reaction times result in only modest gains; in contrast, slag rich in reactive Ca phases benefits more from extended time, allowing continued dissolution before saturation and precipitation limit further extraction. Figure S2B (treatment vs CaO content) suggests that treatment effectiveness increases with CaO content, suggesting that pretreatment primarily acts by exposing reactive Ca-bearing phases rather than forming new ones. Figure S2C (leaching agent vs CaO content) provides the clearest chemical insight: stronger solvent yield the greatest gains when sufficient reactive Ca is present in the feed. Taken together, these plots support the ANOVA result that the extraction route is fundamentally chemistry-controlled, while treatment and time serve as enabling variables that determine how efficiently the reactive calcium can be mobilized.^5,53^

### 3.2 Direct Carbonation

#### 3.2.1 ANN Modeling with Literature Data

For direct carbonation, similarly an 80%, 10%, 10% training, validation, and testing split was used. The final model used yielded an average RMSE of ±0.03206 g of CO_2_-sequestered/g-slag, with an average *R* value of 0.97 for all data indicating a strong agreement between the model predictions and the experimental observations. A graphical representation of the model’s performance is provided in Figure 7.

**Figure 7:**
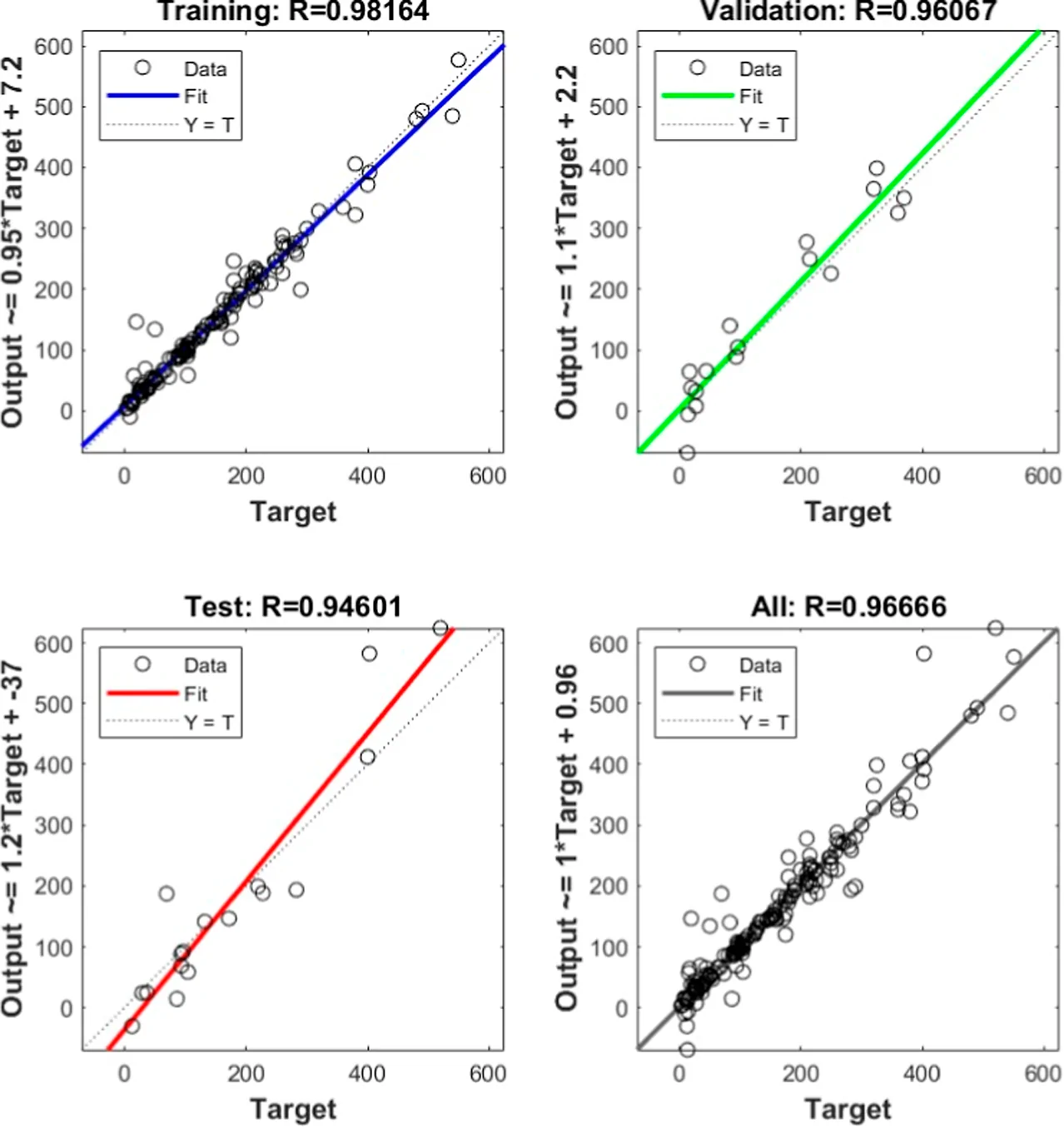
Direct carbonation efficiency ANN analysis results for steel slag data.

Figure 7 shows that the ANN model provides a reasonable prediction of direct carbonation performance, although with higher variability compared to that of leaching. This reflects the inherently complex nature of direct carbonation, which involves simultaneous calcium dissolution, CO_2_ transfer, and carbonate precipitation, all of which introduce nonlinear behavior and variability across studies.^16,18^ Figure 8 indicates that the most influential variables are carbonation assisting agent, CaO content, reaction time, and CO_2_ concentration. The strong influence of assisting agents highlights the importance of solution chemistry in enhancing CO_2_ solubility and carbonate formation, as reported in studies using additives such as bicarbonates and amines.^21,22^ The importance of reaction time reflects the kinetic nature of the process, where carbonation proceeds rapidly initially but slows due to surface passivation by carbonate layers, a well-known limitation in steel slag carbonation.^19^ The moderate influence of CO_2_ concentration further is consistent with gas-phase availability controlling the reaction driving force, while the weaker role of particle size and solid/liquid ratio suggests that mass transfer is not the primary limitation under most conditions.

**Figure 8:**
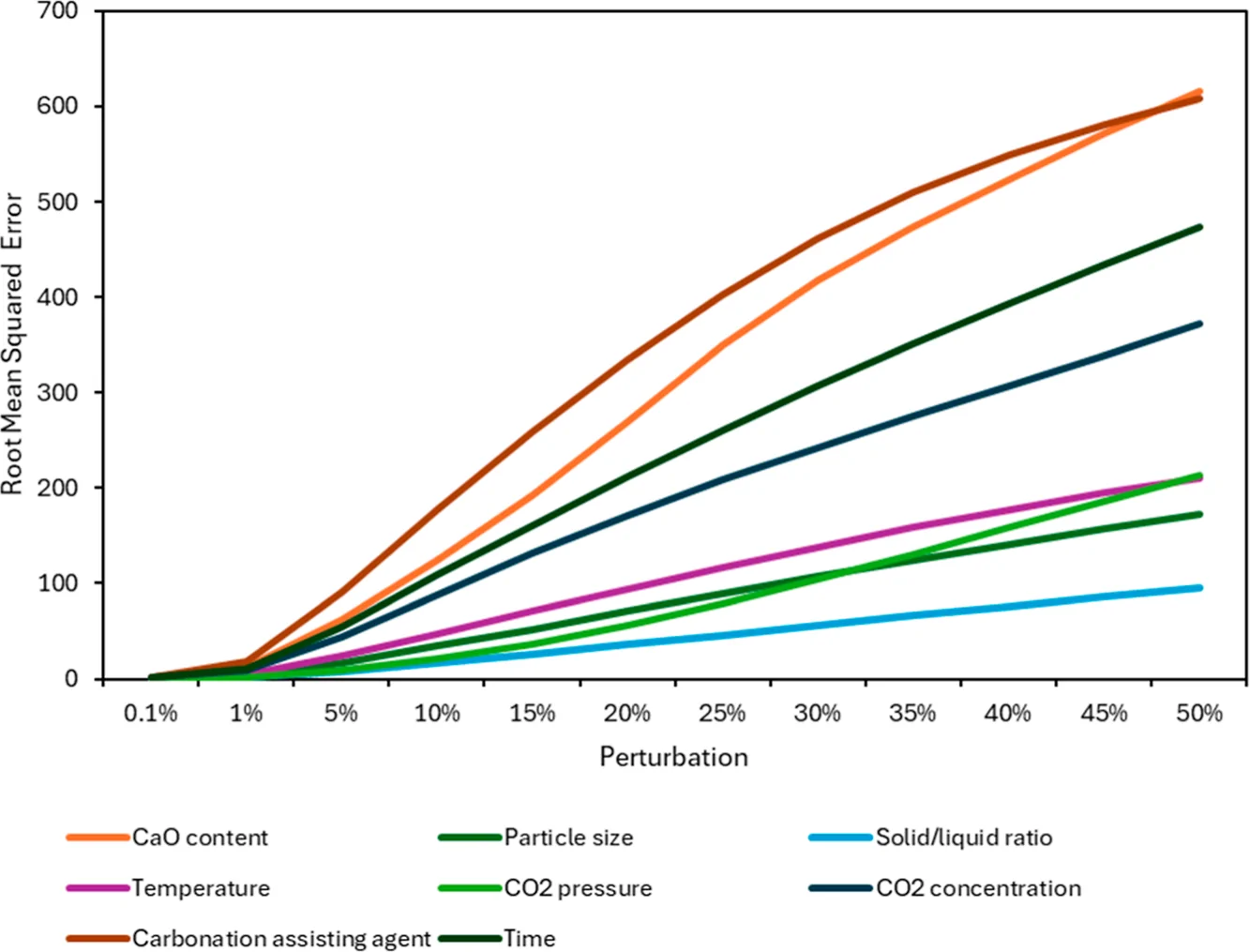
Direct carbonation ANN sensitivity analysis results for steel slags.

#### 3.2.2 3^*k*^ Factorial and ANOVA Results

A similar approach to extraction analysis was used here. The full factorial 3^8^ experimental design using two data sets of 100% and 80% including 6561 virtual experiments was conducted. *F*-Values and *P*-values of ANOVA Statistical Analysis are given in the Supporting Information (Tables S4 and S5). Results are shown in Figures 9 and 10, as bar charts of normalized *F*-values.

**Figure 9:**
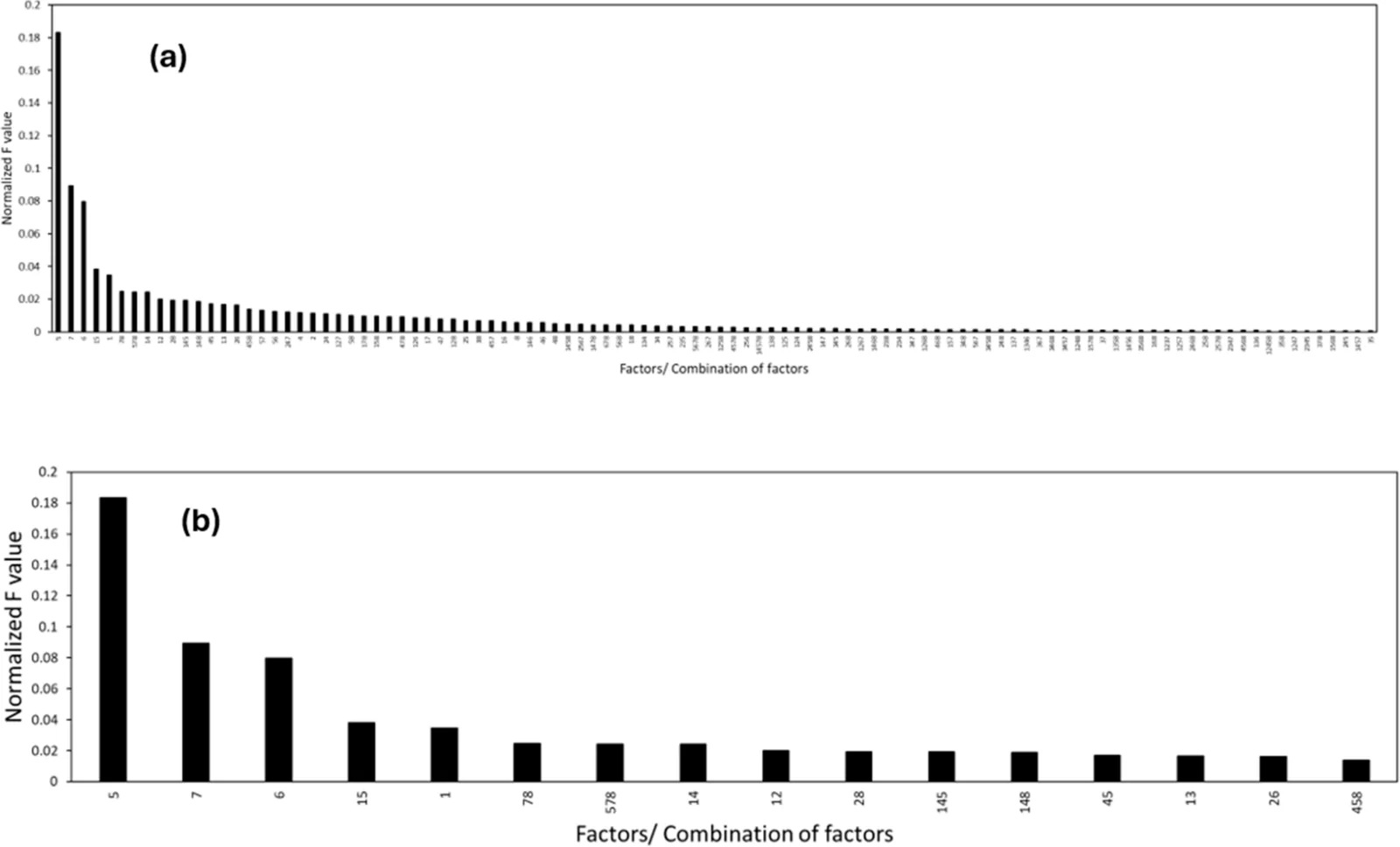
Direct carbonation ANOVA analysis results for steel slags using 100% of data; (a) full ranking, (b) first 15 rankings.

**Figure 10:**
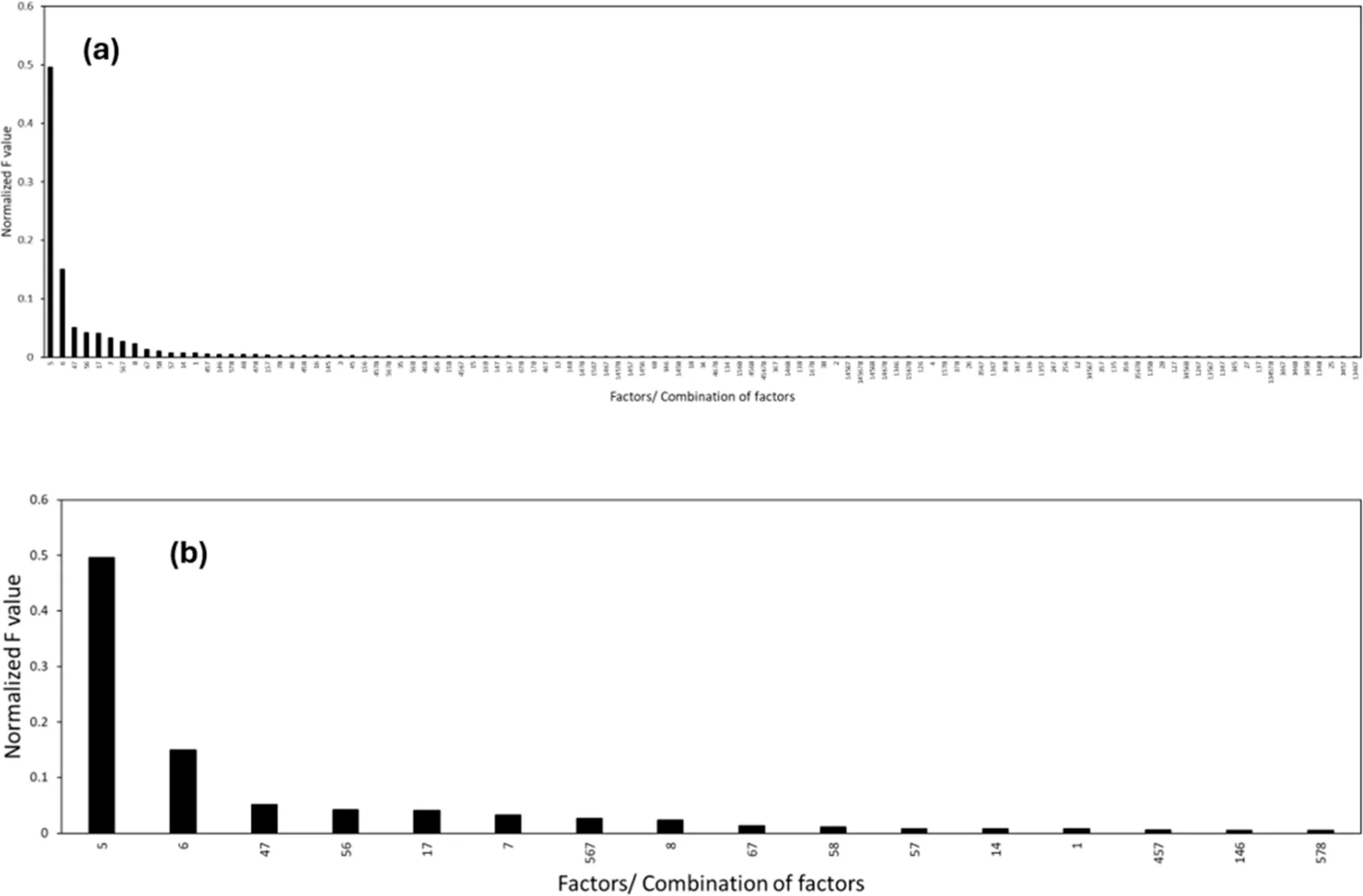
Direct carbonation ANOVA analysis results for steel slags using 80% of data; (a) full ranking, (b) first 15 rankings.

For the 100% direct-carbonation data set, the first 15 ranked terms are 5, 7, 6, 15, 1, 78, 578, 14, 12, 28, 145, 148, 45, 13, 26. The first three positions, CO_2_ pressure (5), carbonation assisting agent (7), and CO_2_ concentration (6), immediately show that direct carbonation is governed by the joint availability of carbon and accessible reactive surface. This is mechanistically sensible because direct aqueous carbonation requires not only a thermodynamic driving force for carbonation but also sufficiently exposed Ca-bearing phases to sustain simultaneous dissolution and precipitation. The fourth and fifth terms, 15 (CaO × CO_2_ pressure) and 1 (CaO content), show that feed chemistry still matters strongly, but its benefit depends on the gas-phase driving force. In other words, a Ca-rich slag cannot fully realize its carbonation potential unless pressure is high enough to enhance dissolved CO_2_ availability. The appearance of 78 (assisting agent × time) and 578 (CO_2_ pressure × assisting agent × time) further indicates that the assisting agent and additive chemistry work together to overcome the classical surface-passivation problem. The next ranks, 14 (CaO × temperature), 12 (CaO × particle size), 28 (particle size × time), 145 (CaO × temperature × CO_2_ pressure), 148 (CaO × temperature × time), 45 (temperature × CO_2_ pressure), 13 (CaO × solid/liquid ratio), and 26 (particle size × CO_2_ concentration), reinforce that direct carbonation is a mixed kinetic–chemical system, where gas availability dominates, but the response surface is shaped by how feed composition, surface accessibility, additives, and thermal conditions interact. This interpretation is strongly aligned with the direct slag-carbonation literature emphasizing coupled roles of CO_2_ partial pressure, accessible labile Ca, additive chemistry, and progressive carbonate-layer formation.^5,7,19,21^

For the 80% direct-carbonation data set, the first 15 terms are 5, 6, 47, 56, 17, 7, 567, 8, 67, 58, 57, 14, 1, 457, 146. This ranking is even more informative because it reveals how the process behaves within the central operating range represented by the literature. CO_2_ pressure (5) and CO_2_ concentration (6) remain dominant, confirming that gas-phase carbon supply is still the strongest lever. But the next ranks are now interactions: 47 (temperature × assisting agent) and 56 (CO_2_ pressure × CO_2_ concentration). This means that once extreme literature values are removed, the direct route is controlled less by raw factor magnitude and more by the synchronization of gas supply, activation, and thermal regime. The presence of 17 (CaO × assisting agent) and 7 (assisting agent) shows that assisting agent matters most when it can unlock a calcium-rich feed. Factor 567 (CO_2_ pressure × CO_2_ concentration × assisting agent) is especially important because it shows that gas availability and surface accessibility are not independent pathways; they must be aligned. The next terms, 8 (time), 67 (CO_2_ concentration × assisting agent), 58 (CO_2_ pressure × time), 57 (CO_2_ pressure × assisting agent), 14 (CaO × temperature), 1 (CaO content), 457 (temperature × CO_2_ pressure × assisting agent), and 146 (CaO × temperature × CO_2_ concentration), indicate that additives, gas-phase forcing, and temperature become powerful only when they act on a sufficiently reactive and exposed solid surface. This indicates that optimization of direct carbonation should focus on combined operating windows rather than single-factor optimization.^7,19,21^

### 3.3 Indirect Carbonation

#### 3.3.1 ANN Modeling with Literature Data

For indirect carbonation, a 50%, 25%, 25% training, validation, and testing split was used due to the lack of sufficient experimental data to maximize the efficiency of the model training and performance. The final model used yielded an average RMSE of ±0.02826 g of CO_2_-sequestered/g-slag, with an average *R* value of 0.87 for all data, indicating a satisfactory agreement between the model predictions and the experimental observations. A graphical representation of the model’s performance is provided in Figure 11.

**Figure 11:**
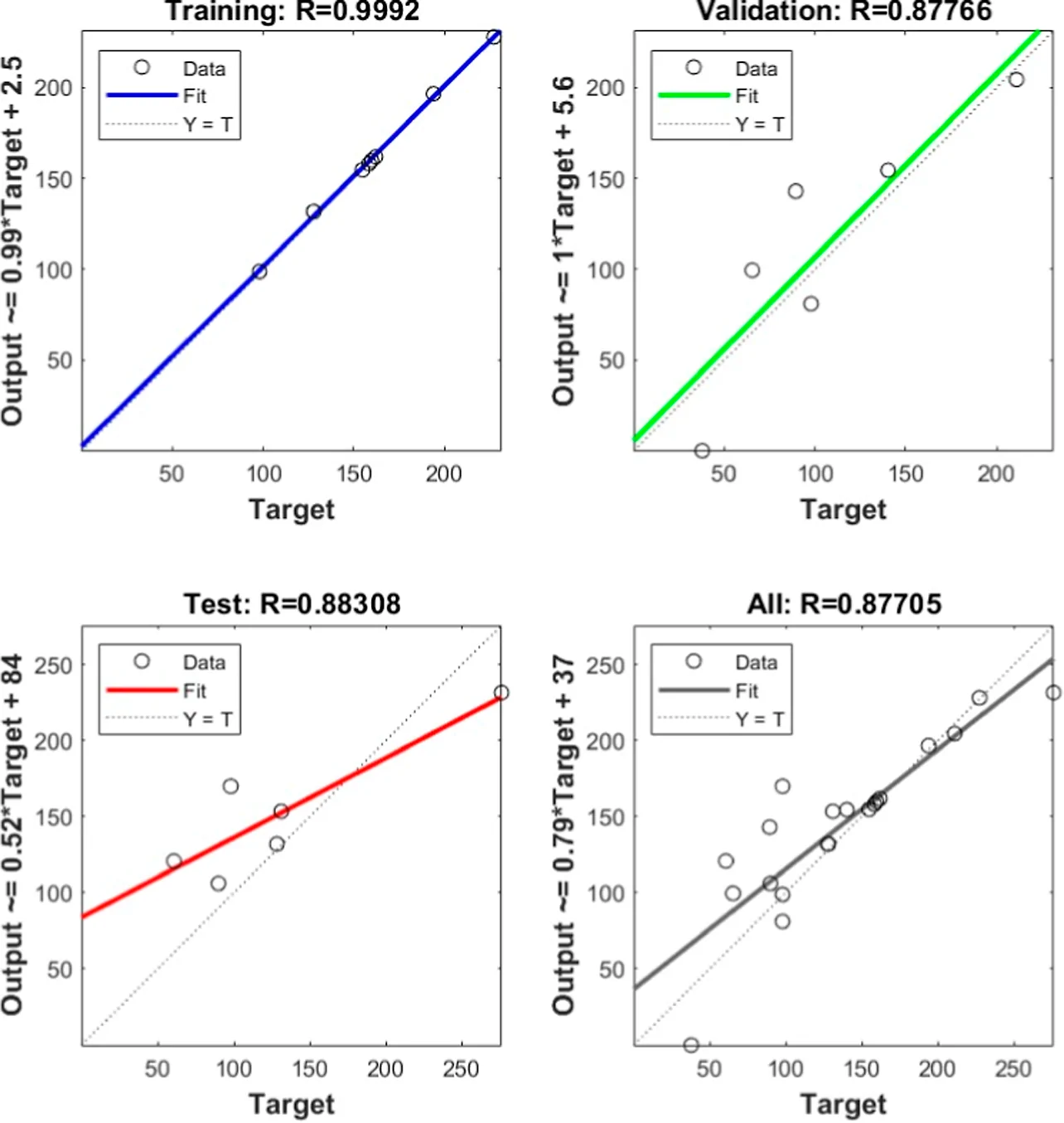
Indirect carbonation efficiency ANN analysis results for steel slag data.

Figure 11 shows that the ANN model for indirect carbonation has lower predictive accuracy, which is expected due to the limited data set size and narrower representation of process conditions. Such limitations are common in data-driven models and should be interpreted cautiously, particularly when extrapolating beyond the training domain.^37^ Figure 12 indicates that the dominant parameters are pH, temperature, CO_2_ concentration, and time, confirming that indirect carbonation is governed by solution-phase chemistry and precipitation kinetics. The strong influence of pH reflects its control over carbonate speciation and precipitation equilibria, while temperature and time influence nucleation and crystal growth rates.^27,28^

**Figure 12:**
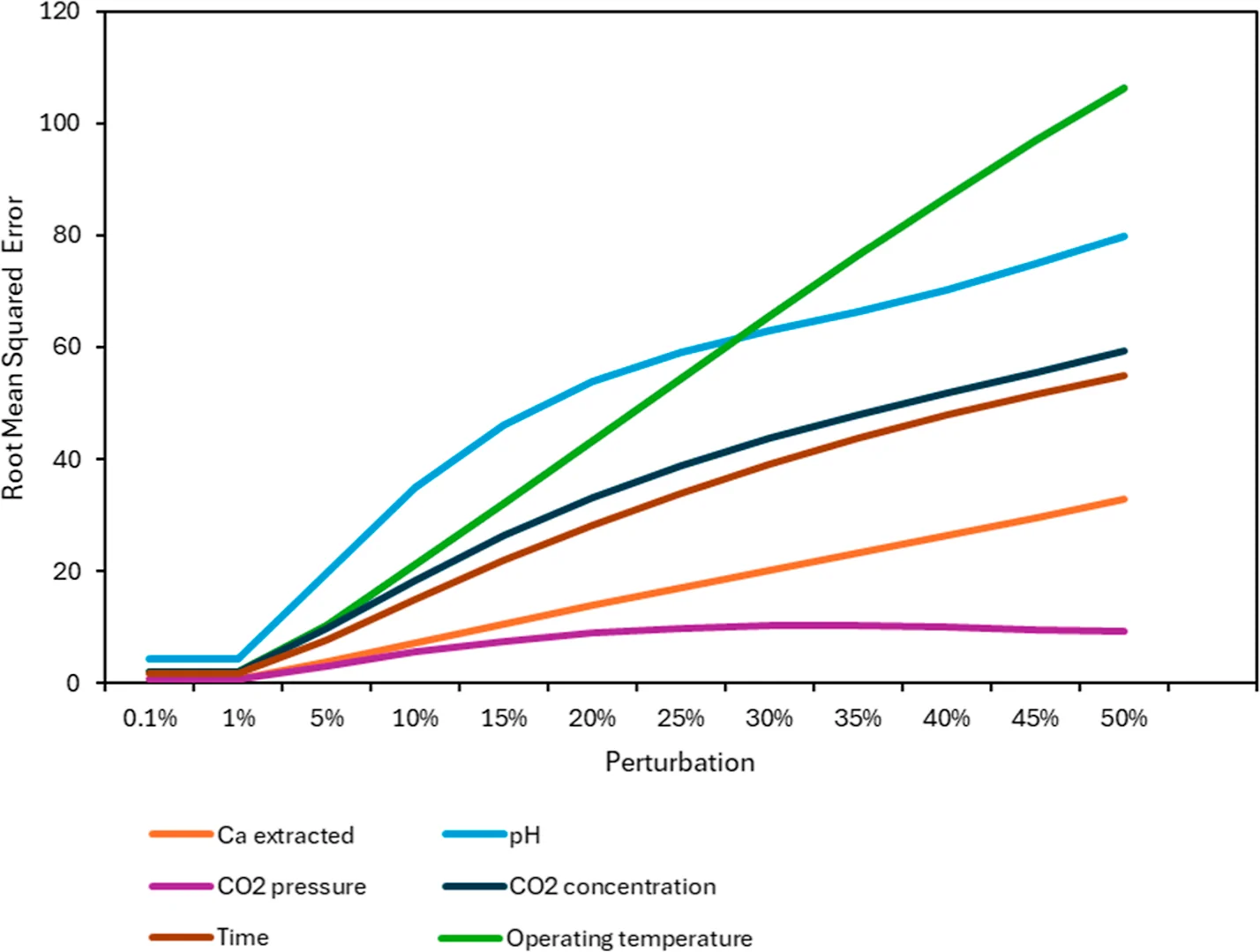
Indirect carbonation ANN sensitivity analysis results for steel slags.

#### 3.3.2 3^*k*^ Factorial and ANOVA Results

A full factorial 3^6^ experimental design was applied, using two data sets (100% and 80%), resulting in 729 virtual experiments. The *F*- and *P*-values from the ANOVA statistical analysis are provided in the Supporting Information (Tables S6 and S7). The results are presented in Figures 13 and 14 as bar charts of normalized *F*-values. Because the indirect-carbonation ANN was trained on a comparatively limited data set, the ANOVA rankings should be interpreted primarily as indications of dominant trends rather than definitive mechanistic proof. The interaction patterns identified here are consistent with established precipitation chemistry, but their relative ranking remains more uncertain than for the leaching and direct-carbonation data sets.

**Figure 13:**
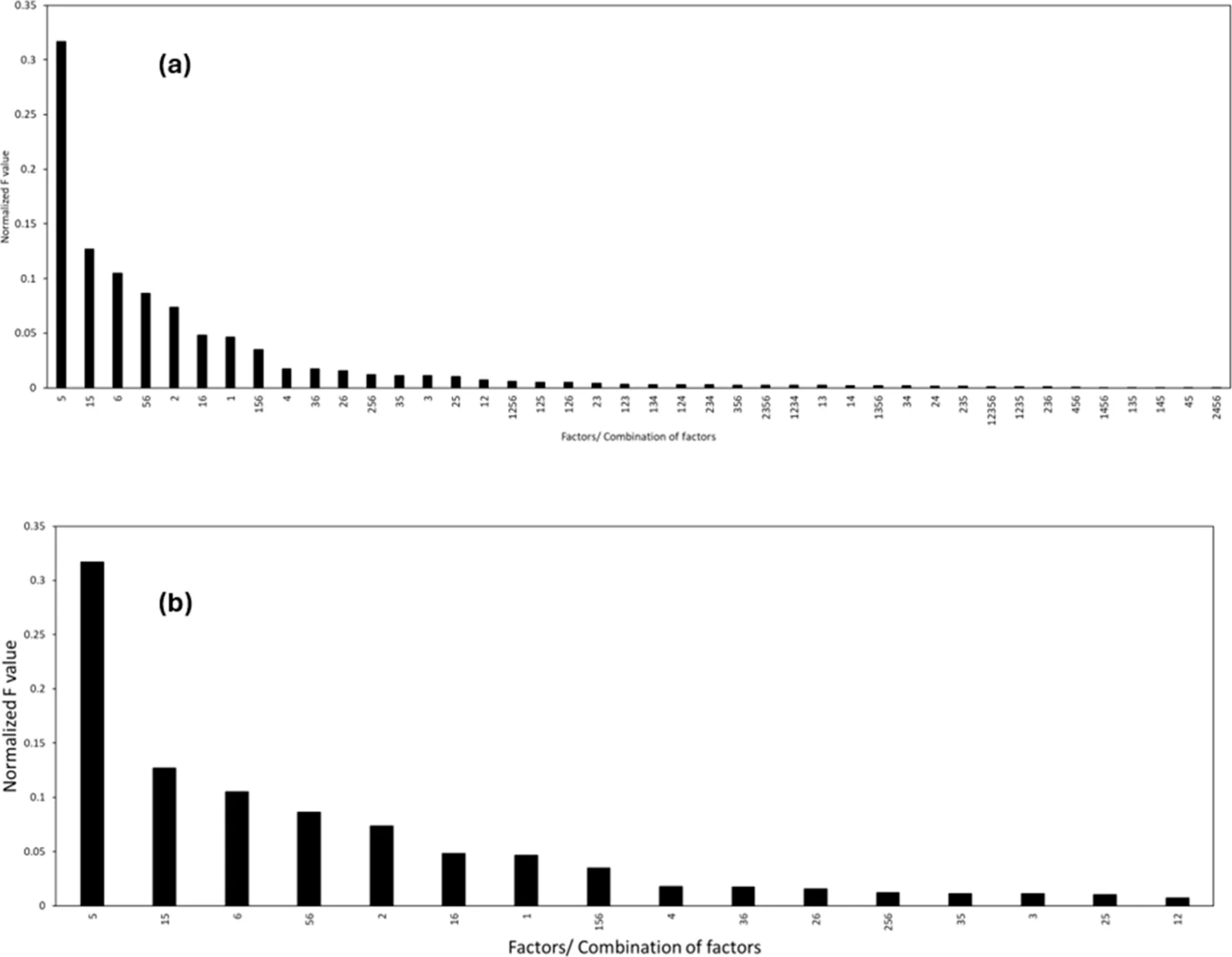
Indirect carbonation ANOVA analysis results for steel slags using 100% of data; (a) full ranking, (b) first 15 rankings.

**Figure 14:**
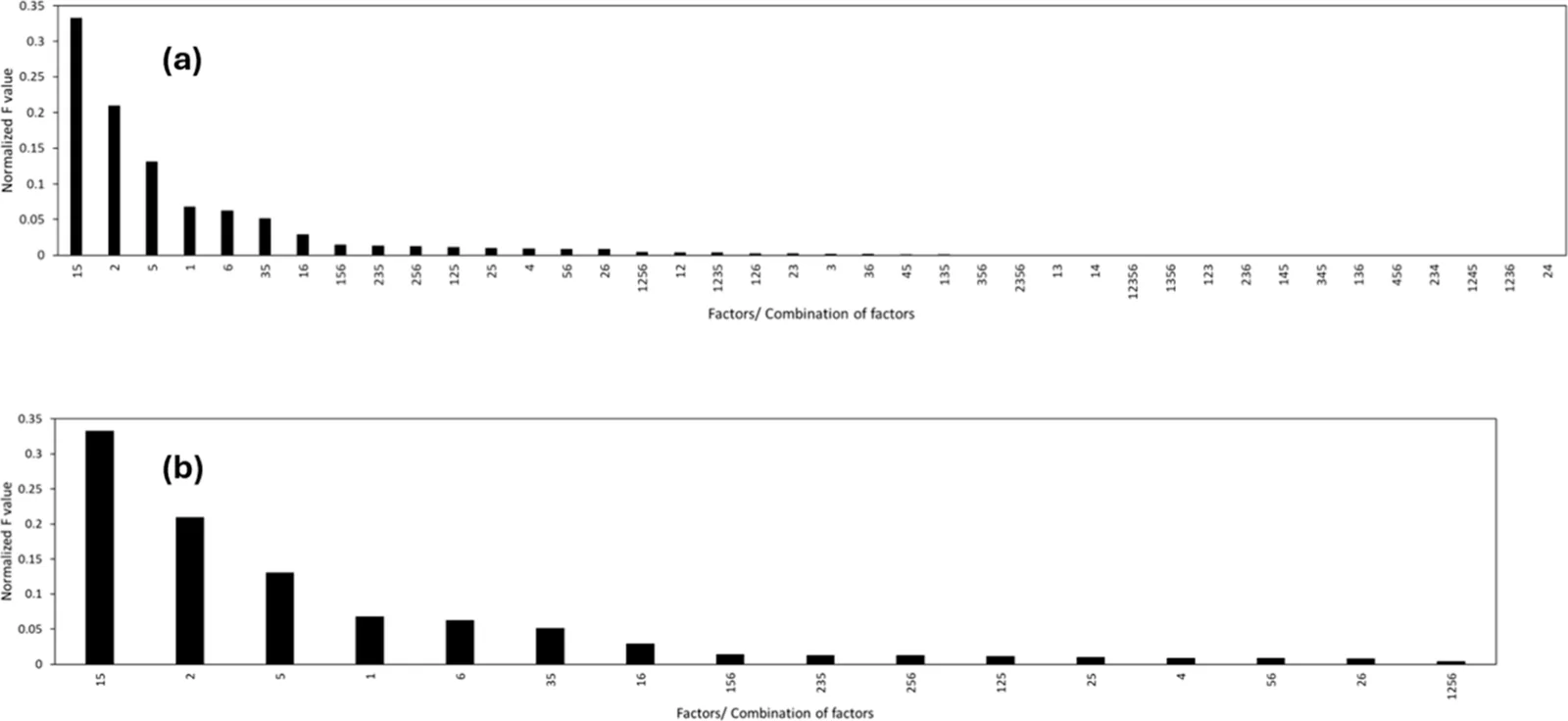
Indirect carbonation ANOVA analysis results for steel slags using 80% of data; (a) full ranking, (b) first 15 rankings.

For the 100% indirect-carbonation data set, the first 15 terms are 5, 15, 6, 56, 2, 16, 1, 156, 4, 36, 26, 256, 35, 3, 25. The strongest term is CO_2_ concentration (5), followed by Ca extracted × CO_2_ concentration (15) and time (6). This is consistent with indirect carbonation behaving as a solution-controlled precipitation process. The dominant role of 15 shows that having a concentrated calcium-rich leachate is not enough by itself; carbonation capacity depends on whether sufficient carbon is delivered to create supersaturation. The strong ranking of 56 (CO_2_ concentration × time) means time has value primarily when carbon supply is maintained. Temperature (2) and 16 (Ca extracted × time) then show that precipitation kinetics and residence time govern how efficiently the dissolved Ca is converted into solid carbonate. The remaining first-15 terms, 1 (Ca extracted), 156, 4 (CO_2_ pressure), 36 (pH × time), 26 (temperature × time), 256, 35 (pH × CO_2_ concentration), 3 (pH), and 25 (temperature × CO_2_ concentration), are all consistent with a precipitation regime in which supersaturation, carbonate speciation, and crystal-growth kinetics are tightly coupled. In short, the 100% indirect-carbonation ANOVA shows that the route is governed first by supersaturation creation, second by kinetic residence time, and third by pH/temperature control of precipitation chemistry.^21,24,54^

For the 80% indirect-carbonation data set, the first 15 terms are 15, 2, 5, 1, 6, 35, 16, 156, 235, 256, 125, 25, 4, 56, 26. Once outliers are removed, the dominant term becomes Ca extracted × CO_2_ concentration (15), which is broadly consistent with expectations for a precipitation-controlled process operating within a narrower practical window. This suggests that the controlling variable may not be governed solely by dissolved Ca or CO_2_ supply, but their joint ability to create and sustain supersaturation. The next four ranks, temperature (2), CO_2_ concentration (5), Ca extracted (1), and time (6), show that kinetic acceleration, carbon availability, dissolved calcium inventory, and residence time together define the window of effective carbonate formation. The next interactions are revealing: 35 (pH × CO_2_ concentration) indicates that carbon delivery is modulated by carbonate speciation; 16 (Ca extracted × time) indicates that richer leachates need sufficient time to fully precipitate; 156 (Ca extracted × CO_2_ concentration × time) shows that the route is intrinsically three-way coupled; 235 and 256 indicate that temperature works jointly with pH and time; 125 and 25 show that temperature and dissolved Ca inventory are linked through precipitation kinetics; 4 (CO_2_ pressure) and 56 (CO_2_ concentration × time) remain relevant but secondary; 26 (temperature × time) completes the picture of an aqueous reaction whose performance is set by supersaturation, pH-dependent speciation, and crystal-growth time rather than by solid-state accessibility. This clearly demonstrates the distinction between indirect and direct carbonations. Indirect carbonation surface plots for steel slags (80% data set) are included in the Supporting Information (Figure S3). Figure S3A shows that the effect of CO_2_ concentration increases at higher temperatures, indicating that temperature not only accelerates the reaction but also improves the efficiency of carbon conversion into carbonate precipitation. Figure S3B shows that the influence of time increases as temperature rises, which is typical of a precipitation-limited system where nucleation and growth become faster once thermal conditions are favorable. These surface plots support the ANOVA results, showing that indirect carbonation is governed by the combined effects of temperature, carbon supply, and reaction time, while pH and dissolved Ca determine the overall supersaturation. More broadly, the contour plots show that the system response is interaction-dependent. The curvature and steepening of the contours indicate that the effect of one variable depends on the level of another, reflecting the synergistic interactions identified by ANOVA. Practically, this indicates that identifying a single optimal temperature, time, or CO_2_ concentration in isolation is less meaningful than defining an operating window in which these variables interact simultaneously. This kind of coupled interpretation is especially important in slag carbonation, where reactive-phase accessibility, carbon supply, and solution chemistry evolve simultaneously during the process.^7,21^

## 4 Other Considerations: Literature Context for the Modeling Results

The following sections do not derive directly from the ANN–factorial framework developed in this study. Instead, they provide contextual interpretation of the modeling results using published literature on steel-slag carbonation, solvent regeneration, process economics, energy consumption, and environmental performance.

### 4.1 Calcium Carbonate Phase Formation and Product Characteristics

The mineralization of CO_2_ in steel slag systems results in the formation of calcium carbonate polymorphs, primarily calcite, aragonite, and vaterite, depending on the process conditions such as temperature, pH, and supersaturation. Among these, calcite is the most thermodynamically stable phase, while vaterite and aragonite are metastable and may transform into calcite over time.^15,16,19,55^

In direct carbonation systems, rapid reaction kinetics and high supersaturation conditions often favor the formation of metastable phases, such as vaterite, particularly at early reaction times. As the reaction progresses, these phases tend to transform into calcite, especially under elevated-temperature conditions. This transformation behavior is consistent with studies showing that carbonation time and temperature significantly influence phase evolution and crystallinity.^21,27^

In indirect carbonation, where precipitation occurs in solution, the pH and supersaturation play a dominant role in determining polymorph formation. High-pH conditions promote rapid nucleation, which can initially favor vaterite formation, while controlled supersaturation conditions enable the formation of larger and more stable calcite crystals. This suggests that indirect carbonation offers greater flexibility for tailoring product characteristics, which may be advantageous for applications requiring specific CaCO_3_ morphologies.^15,28^

The ability to control carbonate-phase composition is particularly important for potential valorization of the products in construction materials, fillers, or industrial applications, where particle size, morphology, and purity are critical performance parameters.

### 4.2 Solvent Regeneration and Process Integration

Solvent or extractant regeneration is one of the clearest process-level differences between direct and indirect carbonations. In the direct route, additives such as MEA, NaHCO_3_, and K_2_CO_3_ mainly function as carbonation-assisting agents that improve CO_2_ dissolution, carbonate speciation, or calcium release, but they do not usually define the entire process economics, unless they must be recovered or replenished repeatedly. In contrast, in the indirect route, the extractant is part of the core process architecture, and its recoverability largely determines whether the process remains technically and economically attractive.^5,21^

For strong mineral acids such as HCl, high calcium extraction efficiencies can be achieved; for instance, Lee et al.^53^ reported up to 97% Ca extraction from steel slag using 2 M HCl, although significant coextraction of Mg and Al was also observed, highlighting the inherent trade-off between extraction efficiency and selectivity. Acid systems are effective, but unless acid is regenerated through downstream chemical recovery, they impose a high reagent burden and additional neutralization demand.^56^ This is one reason why strong acid routes are often more attractive for laboratory extraction studies than for full circular process design.^57^

Ammonium salts are attractive because they can offer better selectivity and, in several process concepts, can be regenerated after carbonation. Recent work on blast-furnace slag with (NH_4_)_2_SO_4_ showed that the extractant is recyclable and that under a roasting-assisted route, nearly complete extraction of Ca, Mg, and Al could be achieved under optimized conditions, with approximately 99% Ca and 89% Mg eventually converted into carbonate products.^58^ The same study reports that part of the ammonia can be recovered through subsequent decomposition steps, which is important because ammonia regeneration is central to closing the reagent loop. Likewise, ammonium-based systems are widely recognized as advantageous because they function as recyclable proton carriers, in contrast to strong acids that are typically consumed; this recyclability is a key factor enabling more sustainable mineral carbonation routes.^59^ Consistent with this, reviews of steel-slag carbonation processes note that ammonium salts are appealing specifically because they can be recycled and can produce relatively pure carbonate products compared with strong acid routes.^5^ For NH_4_Cl, indirect steel-slag carbonation studies have reported that carbonation of the calcium-rich leachate can regenerate NH_4_Cl while producing high-purity CaCO_3_. For example, Sun et al.^54^ reported the production of *ca.* 96 wt % CaCO_3_ under optimized carbonation conditions, with only minor Mg-containing impurities remaining. More recent studies continue to emphasize NH_4_Cl as an attractive lixiviant due to its combined Ca selectivity and potential recyclability, although practical implementation highlights that extraction efficiency depends on operating conditions and that long-term reagent recovery and losses remain important considerations for scale-up.^60^

Organic acids, particularly acetic acid, are frequently discussed because they can selectively dissolve calcium while operating under milder conditions than those of strong mineral acids. They are attractive in integrated indirect carbonation or Ca sorbent preparation schemes, but practical deployment depends on whether the acid can be economically recovered from the postleaching solution. Early work by Eloneva et al.^61^ demonstrated that acetic acid can selectively extract calcium from steelmaking slag, enabling the subsequent precipitation of relatively pure CaCO_3_. However, their subsequent process analyses have shown that chemical consumption and regeneration requirements (e.g., NaOH use or solvent recovery) can dominate the overall process cost and feasibility at scale.^62^ More recent slag-based work using acetic acid for sorbent synthesis and downstream steam regeneration further emphasizes that process integration, particularly heat management and recyclability, becomes critical when moving beyond laboratory scale, with acid recovery and thermal regeneration emerging as key determinants of overall performance.^63^

Amines deserve separate consideration, as they are typically used as CO_2_ capture and mineralization enhancers rather than primary calcium lixiviants. In aqueous systems, amines react with CO_2_ to form carbamate and bicarbonate species, thereby increasing dissolved inorganic carbon and accelerating subsequent mineral carbonation reactions.^64,65^ While amines are effective for enhancing reaction rates and enabling coupled CO_2_ capture–mineralization pathways, they are not inherently advantageous from an energy or cost perspective unless regeneration is carefully integrated (e.g., with low-grade heat or advanced regeneration strategies such as solid-supported amines).^66^ These considerations are consistent with broader reviews of slag carbonation systems, which emphasize that additive-based enhancements such as amines must be evaluated within the full process context rather than solely on their kinetic benefits.^5,24^

From a process design perspective, key criteria for solvent regeneration include extraction selectivity, reagent loss, regeneration energy demand, carbonate purity, and compatibility with process integration (e.g., waste heat utilization). These factors also align with our ANN results for indirect carbonation, which indicate that the process performance is highly sensitive to solution chemistry; consequently, the value of a solvent depends not only on calcium extraction efficiency but also on how effectively and economically it can be regenerated.

### 4.3 Environmental Implications and Heavy Metal Stabilization

From an environmental perspective, steel-slag carbonation offers dual benefits by permanently sequestering CO_2_ while diverting alkaline waste from landfill. However, overall performance depends not only on CO_2_ uptake but also on the extent to which carbonated products displace conventional materials, the energy required for preprocessing and carbonation, and the impact on trace-metal mobility.^5,16,67^ A key difference between direct and indirect routes lies in where the environmental burden occurs. In direct carbonation, major hotspots typically include feedstock grinding, CO_2_ capture/compression, and thermal activation, which contribute significantly to overall energy demand. The main advantage of direct routes is their relative process simplicity and low chemical demand, although incomplete conversion and lower product purity can limit the value obtained per unit of energy or CO_2_ processed.^68–70^ In contrast, indirect carbonation can produce higher-purity carbonate products and improved conversion, but shifts the environmental burden toward solvent production, solvent regeneration, liquid handling, and downstream separations, which often dominate cost and energy use if not efficiently integrated.^57^ In other words, indirect routes tend to perform better in terms of product quality, but risk burden shifting into energy and reagent consumption, particularly when solvent regeneration is inefficient or poorly integrated within the process.^71,72^

Published life cycle assessments highlight a trade-off between the environmental benefits and drawbacks of CO_2_ recovery and mineral carbonation, largely driven by energy use and reagent demand. For example, a study on steel-slag carbonation, as a CCUS-based route, showed the reduced the global warming potential by up to 480% compared to conventional hot-braising steel slag treatment, achieving −76.78 kg CO_2_-equiv per tonne of slag.^67^ This large relative reduction arises because the carbonation route both sequesters CO_2_ and avoids emissions associated with conventional thermal stabilization, whereas the hot-braising route is energy-intensive and emission-positive. More broadly, LCA studies of mineral carbonation consistently show that, although carbonation can substantially reduce climate impacts, particularly when carbonated products displace conventional materials, energy demand remains the dominant environmental hotspot, especially for preprocessing and CO_2_ handling.^68^ Heavy-metal behavior is another important environmental distinction with steel slags.^16^ Carbonation can reduce leaching of some trace elements by converting free lime and unstable phases into more stable carbonates and by physically encapsulating metals in newly formed matrices.^19,73^ Leaching studies on carbonated steel slags show that the transformation of Ca-bearing phases (e.g., portlandite and silicates) into calcite and the increased presence of Fe/Al hydroxides can decrease the mobility of several sorption-controlled trace elements.^19,74^ Reviews and experimental studies therefore often describe carbonation as both a CO_2_ sequestration route and a stabilization treatment, improving volume stability while reducing the release of certain contaminants.^16^ However, this benefit is not universal: changes in speciation during carbonation can increase the leaching of specific elements such as vanadium, and concerns related to chromium and other oxyanion-forming species may persist under certain conditions.^15,16,19^ That is why a defensible environmental assessment should explicitly consider both CO_2_ sequestration performance and trace-element behavior, rather than focusing on carbon storage alone.

### 4.4 Energy Consumption and Process Efficiency

Energy consumption represents one of the most important distinctions between direct and indirect carbonation pathways. Although both routes ultimately convert alkaline phases into stable carbonate minerals, the distribution of energy demand and the mechanisms controlling process efficiency differ substantially. For direct aqueous carbonation, the primary energy requirements are associated with feedstock and slurry preparation, reactor heating, and CO_2_ compression or pressurization; for instance, Costa et al.^75^ reported energy requirements of 980–6300 MJ t^–1^ CO_2_ for slurry-phase (direct aqueous) carbonation of BOF slag, with energy demand primarily associated with grinding, slurry handling, heating, CO_2_ compression, and solid/liquid separation. Previous process analyses have shown that grinding and high-pressure operation account for a significant fraction of total process energy demand, particularly when fine particle sizes (<100 μm) and elevated CO_2_ pressures (>10 bar) are employed to accelerate carbonation kinetics.^76,77^ The benefit of these energy inputs is improved calcium accessibility and enhanced carbonation rates. For example, Huijgen et al.^18^ reported carbonation extents of up to ∼74% of available Ca within 30 min in aqueous systems, corresponding to CO_2_ uptakes approaching 0.18–0.25 t CO_2_ per tonne of steel slag depending on slag composition. More recent studies have reported carbonation efficiencies ranging from approximately 50–90% and CO_2_ sequestration capacities of 100–300 kg CO_2_ t^–1^ slag under optimized laboratory conditions.^7,16,78,79^ These relatively high reaction rates are attributable to the intrinsic reactivity of steel slag, which contains readily available Ca-bearing phases such as free CaO, C_2_S, and C_3_S. As a result, direct carbonation can achieve substantial CO_2_ uptake without requiring chemical extraction and solvent-regeneration steps, making it attractive from a process-simplicity perspective. Indirect carbonation follows a fundamentally different energy distribution. While requirements for high-pressure carbonation and extensive grinding may be reduced, substantial energy is instead consumed during calcium extraction, liquid/solid separation, solution conditioning, solvent regeneration, evaporation, and reagent recovery.^57^ The extraction stage can achieve calcium recoveries exceeding 90%, depending on the solvent employed, which is generally higher than what is attainable through direct carbonation pathways.^28^ However, these gains are accompanied by additional energy and operational penalties. Published studies indicate that solvent regeneration and chemical recovery frequently become the dominant contributors to both operating cost and process energy consumption, particularly in systems employing strong acids or alkali absorbents, with acid–base recycling identified as essential for process viability and capable of reducing costs compared to nonregenerated systems.^57,80^

### 4.5 Economic Considerations and Industrial Feasibility

The economic feasibility of steel slag carbonation depends strongly on process route, system boundary, reagent recovery, CO_2_ source, energy demand, and product value. Direct and indirect carbonation should therefore not be compared using conversion efficiency alone. Instead, the relevant economic question is whether the additional processing required to improve conversion, or product purity can be justified by lower net CO_2_ cost, reduced waste-disposal burden, or higher-value carbonate products. For direct aqueous carbonation, Huijgen et al.^77^ conducted a techno-economic benchmark study, estimating a sequestration cost of approximately €77/t CO_2_ net avoided for steel slag, compared with €102/t CO_2_ net avoided for wollastonite. The lower cost for steel slag was attributed mainly to the absence of feedstock mining costs, since steel slag is an industrial residue generally available near steelmaking sites. In the same analysis, major cost drivers were associated with grinding, CO_2_ compression, and reactor operation (200 °C and 20 bar), while the liquid-to-solid ratio and potential value of the carbonated product were identified as important sensitivity parameters. Sanna et al.^81^ reported that the cost of direct aqueous carbonation of inorganic wastes, including concrete waste and steel slag, ranges from approximately 8–104 U.S. dollars per t-CO_2_, depending on operating conditions such as reactor configuration (e.g., spray or trickle-bed systems), temperature, pressure, CO_2_ concentration, and the extent of product valorization. Indirect carbonation has a different cost structure. Although it can improve calcium utilization and produce higher-purity precipitated calcium carbonate (PCC), it introduces additional costs for extraction, solid/liquid separation, pH adjustment, solvent recovery, and reagent regeneration. In the absence of reagent recycling, indirect aqueous carbonation routes designed to produce high-purity PCC or hydromagnesite from alkaline residues, including EAF slags or serpentine, have been estimated to require chemical costs of approximately 600–4500 U.S. dollars/t CO_2_ when acids and bases such as HCl, HNO_3_, CH_3_COOH, and NaOH are not regenerated.^81^ The same review also noted that an HCl extraction route evaluated by IEA GHG resulted in an estimated cost of approximately 205 U.S. dollars/t CO_2_ avoided, while chemical regeneration system (e.g., HCl/HNO_3_ with NaOH) can generate CO_2_ emissions equivalent to 2.6–3.5 times the amount sequestered, effectively offsetting the process benefits. Lee et al.^82^ investigated an indirect pH-swing mineralization process, in which calcium is extracted from steel slag via acid leaching and subsequently precipitated as high-purity nano-CaCO_3_ using electrochemically generated acid and base streams. The techno-economic analysis reported a production cost of approximately 484 U.S. dollars/t-CaCO_3_, reflecting the significant energy and capital requirements associated with electrolysis-driven reagent generation. These findings indicate that reagent recovery is not a secondary consideration in indirect carbonation, but rather a critical factor governing both economic feasibility and net carbon performance. Techno-economic studies of related indirect carbonation systems reinforce this conclusion. Proaño et al.^24^ evaluated indirect carbonation for CO_2_ capture in the cement industry and reported estimated capture costs of approximately 65 U.S. dollars/t CO_2_ for a NaOH-based route and 140 U.S. dollars/t CO_2_ for a Ba-based route. Although this study was not specific to steel slag, it is relevant because it suggests that indirect carbonation can be technically feasible when operated under controlled alkaline conditions, but profitability depends strongly on absorbent recovery, carbonate demand, and policy conditions. Similarly, Gomes et al.^57^ showed for pH-swing mineralization of mining wastes that process costs exceed 3000 U.S. dollars/t CO_2_ without acid–base recycling, while internal acid regeneration can reduce costs to approximately 500–800 U.S. dollars/t CO_2_. Under a 10% reagent makeup assumption, costs remained in the range of approximately 800–1800 U.S. dollars/t CO_2_, with carbonate precipitation contributing 34–78% of total costs. These values are not steel-slag-specific, but they are highly relevant to indirect slag carbonation because they demonstrate the dominant economic role of reagent recovery and precipitation-stage design. Product valorization can partly offset these costs. Indirect carbonation is economically attractive when it produces high-purity PCC or other marketable carbonate products rather than only low-value carbonated residues. For example, Kazanç et al.^72^ reported a levelized net profit of approximately 328.4 U.S. dollars/tonne of CO_2_ utilized for a mineralization process producing PCC and ammonium sulfate from flue gas desulfurization gypsum, with a global warming impact approximately 64% lower than conventional production. Although this feedstock differs from steel slag, the result illustrates a broader economic principle: mineral carbonation becomes more competitive when CO_2_ storage is coupled with high-value coproduct generation and heat integration. For steel slags, this suggests that indirect carbonation may be economically justified only when solvent regeneration, product purity, and market value are considered together.

Overall, direct carbonation is generally favored by process simplicity, lower reagent demand, and easier integration at steel-making or slag-processing sites. Its economics are governed mainly by grinding, CO_2_ delivery/compression, reactor operation, and the potential use of carbonated slag as a construction material or a supplementary cementitious product. Indirect carbonation is more chemically intensive and can become costly if acids or bases are consumed stoichiometrically; however, it can become more attractive when high-purity PCC, recyclable extractants, waste heat integration, or coproduct markets offset the additional processing costs. Therefore, the economic comparison between direct and indirect steel slag mineralization should be framed not as a universal cost ranking but as a system-specific trade-off among process simplicity, reagent recovery, product value, and CO_2_ utilization efficiency.

### 4.6 Comparison with Magnesium-Based Feedstocks

The results from this steel slag study can be compared with our previous work on magnesium-rich serpentine wastes.^29^ Both studies used the same ANN–factorial–ANOVA method. Because of this, the comparison is consistent, and differences mainly come from the chemistry of Ca and Mg systems. For the extraction stage, both systems show that chemical effects are more important than physical factors. In the Mg system, pretreatment was the most important factor in the 80% data range. This was followed by extraction agent, temperature, and particle size. The strongest interaction was between MgO content and extraction agent. This result reflects the stable structure of serpentine, which needs strong pretreatment to release Mg. In the steel slag system, the main factors are different. Feed chemistry (CaO content) and leaching conditions are more important. The strongest interaction is between treatment and leaching agent. This difference comes from the mineral structure. Calcium in slag exists in reactive phases such as free CaO and Ca–silicates. In contrast, Mg in serpentine is tightly bound in a stable structure. In both systems, particle size and reaction time are less important. They act as supporting factors, not main drivers.

For direct carbonation, both studies show that the process depends on gas supply, reaction kinetics, and chemistry. In the Mg system, pretreatment was the most important factor. CO_2_ concentration was the second most important, followed by particle size. Solution chemistry (such as pH and additives) had a smaller effect. In the steel slag system, gas-phase factors are more important. CO_2_ pressure and CO_2_ concentration show strong effects, together with Ca-related interactions. Pretreatment is still important, but not as dominant as in the Mg system. This happens because slag already contains reactive Ca phases. Therefore, less activation is needed, and gas supply becomes more important. In both systems, solution pH and additives have a smaller effect in direct carbonation. The largest difference appears in the indirect carbonation stage. In the Mg system, solution chemistry is dominant. The carbonation agent and solution pH are the most important factors. The interaction between these two is also very strong. Other factors such as Mg concentration and CO_2_ pressure have only moderate effects, and temperature has little influence. In the steel slag system, the ranking is different. The strongest effect is the interaction between dissolved Ca concentration and CO_2_ concentration. Temperature, time, and pH interactions are also important. Both systems show that indirect carbonation is controlled by solution chemistry. However, the controlling mechanism is different. In Mg systems, performance is mainly controlled by agent–pH effects. In Ca systems, it is controlled by the balance between dissolved Ca and available CO_2_. This difference is consistent with precipitation chemistry. Magnesium carbonates require specific pH conditions and often need additives to form. Calcium carbonate forms more easily when Ca^2+^ and carbonate ions are available at the same time.

Overall, this comparison shows that mineralization performance depends on multiple factors acting together. No single parameter controls the process. For both Ca- and Mg-based systems, extraction and direct carbonation depend mainly on feed reactivity and gas access. Indirect carbonation is controlled by solution chemistry, but the key controlling factors differ between systems. This also shows that the ANN–factorial method can be applied to different feedstocks. However, the important parameters must be identified separately for each system.

## 5 Conclusion and Practical Implications

This study developed an integrated ANN–factorial framework to analyze CO_2_ mineralization of steel slag through leaching, direct carbonation, and indirect carbonation. The approach provides both prediction capability and clear identification of important variables and their interactions. The results show that each stage of the process is controlled by different mechanisms: leaching is mainly controlled by feed composition, direct carbonation is controlled by both feed properties and CO_2_ supply, and indirect carbonation is controlled by solution chemistry.

For the leaching stage, the most important factors are CaO content, leaching agent, and treatment method. Their interactions, especially treatment × leaching agent, are also very important. This shows that calcium extraction depends mainly on reactive calcium in the feed and chemical activation. Physical factors such as particle size and time have a smaller effect and mainly help the reaction to proceed. Therefore, process design should start with feed characterization and solvent selection, followed by suitable activation methods.

For direct carbonation, the main factors are CO_2_ pressure, CO_2_ concentration, assisting agents, and Ca-related interactions. This is consistent with direct carbonation depends on both CO_2_ availability and accessible reactive calcium. The process behaves as a coupled transport–reaction–chemical system. The results also show that interactions between variables are strong in realistic conditions. Therefore, optimization should focus on combined operating conditions rather than single parameters. In practice, direct carbonation is suitable for simpler systems where moderate conversion and lower complexity are acceptable.

For indirect carbonation, the dominant factors are linked to solution chemistry and precipitation behavior. The strongest effect comes from the interaction between dissolved Ca concentration and CO_2_ concentration, followed by temperature, time, and pH-related effects. This shows that indirect carbonation is controlled by supersaturation and precipitation kinetics. Improving extraction alone is not enough; the carbonation step must also be optimized. Thus, indirect carbonation should be designed as an integrated extraction–precipitation system. This route is more complex but has higher potential for producing high-purity carbonate products.

Comparison with our previously developed Mg-rich serpentine system^29^ further highlights the material-specific nature of mineral carbonation. Although both Ca- and Mg-rich systems are controlled by interacting chemical and kinetic variables, the dominant mechanisms differ because of feedstock mineralogy. Mg-rich serpentine requires stronger pretreatment to liberate structurally bound Mg and shows greater sensitivity to pH and carbonation additives during indirect carbonation. In contrast, steel slag contains more readily reactive Ca-bearing phases, making Ca availability, CO_2_ supply, and Ca–carbonate supersaturation the dominant controls. This comparison suggests that the ANN–factorial framework can be transferred across alkaline feedstocks but that optimization strategies must be developed separately for each mineral system.

Overall, the results show that chemical and kinetic interactions are more important than physical factors, especially under realistic conditions. This explains why one-factor-at-a-time methods are not sufficient for these systems. The ANN–factorial framework provides a better way to understand and optimize the process. However, while the present ANN factorial framework demonstrates strong predictive performance and identifies consistent dominant factors across the leaching, direct-carbonation, and indirect-carbonation routes, several complementary validation analyses are still in progress and will be reported in a future revision or follow-up study. These include publication-grouped cross-validation to more rigorously test generalization to unseen studies and slag sources, a sensitivity analysis of nonordinal categorical encoding, bootstrap-based confidence intervals and rank-stability estimates for the dominant factorial effects, and a sensitivity check excluding the hypothetical intermediate auxiliary-treatment level. We expect these analyses to refine the confidence bounds around the reported associations rather than to alter the principal conclusions, but we present them here as planned extensions of this work.

From a practical point of view, the trained ANN models can be used to construct a virtual experimental space and to support the preliminary screening of operating conditions within the chemical and process domain represented by the literature database. Predictions for new slag sources should therefore be interpreted as guidance for experimental prioritization rather than as direct substitutes for validation experiments. This is particularly important for slags with mineralogy, free CaO content, cooling history, Fe-bearing phases, or reactor conditions that differ substantially from the training data.

## Supplementary Material



## Notes

Literature search and study-selection flow diagram showing the screening of the initial literature pool and selection of study records used for ANN model development; contour plots of interaction between critical affecting factors on calcium extraction efficiency, created with 80% data used for modeling; contour plots of interaction between critical factors affecting indirect CO_2_ mineralization capacity, created with 80% data used for modeling; article library used for detailed analysis; results for ANOVA statistical analysis; final ANN architecture and training controls used for the three process models; and simplified-ANOVA model specification and residual structure (PDF)

## †

Department of Chemical Engineering, University of Waterloo, Waterloo, Ontario N2L 3G1, Canada
