# Supplementary material for: Machine Learning-Driven Design of Experiments for CO2 Mineralization of Steel Slag Materials
Source: ACS Omega. 2026 Oct 19;11(43):67000–21. doi: 10.1021/acsomega.6c07267 (PMC13736851; doi:10.1021/acsomega.6c07267)
Supplement: Supplementary file 1 [file ao6c07267_si_001.pdf]

Machine Learning-Driven Design of Experiments for CO_2 Mineralization of Steel Slag Materials

Hamid Radfarnia, Iris Samputu[†], Kourosh Zanganeh*

Natural Resources Canada, CanmetENERGY, 1 Haanel Drive, Ottawa, Ontario K1A 1M1, Canada

* Corresponding author

E-mail: hamid.radfarnia@nrcan-rncan.gc.ca

Keywords: CO_2 Mineralization, CO_2 sequestration, direct carbonation, indirect carbonation, mineral extraction, factorial design, ANN, steel slags

[†] Present address: Department of Chemical Engineering, University of Waterloo, Waterloo, Ontario N2L 3G1, Canada.

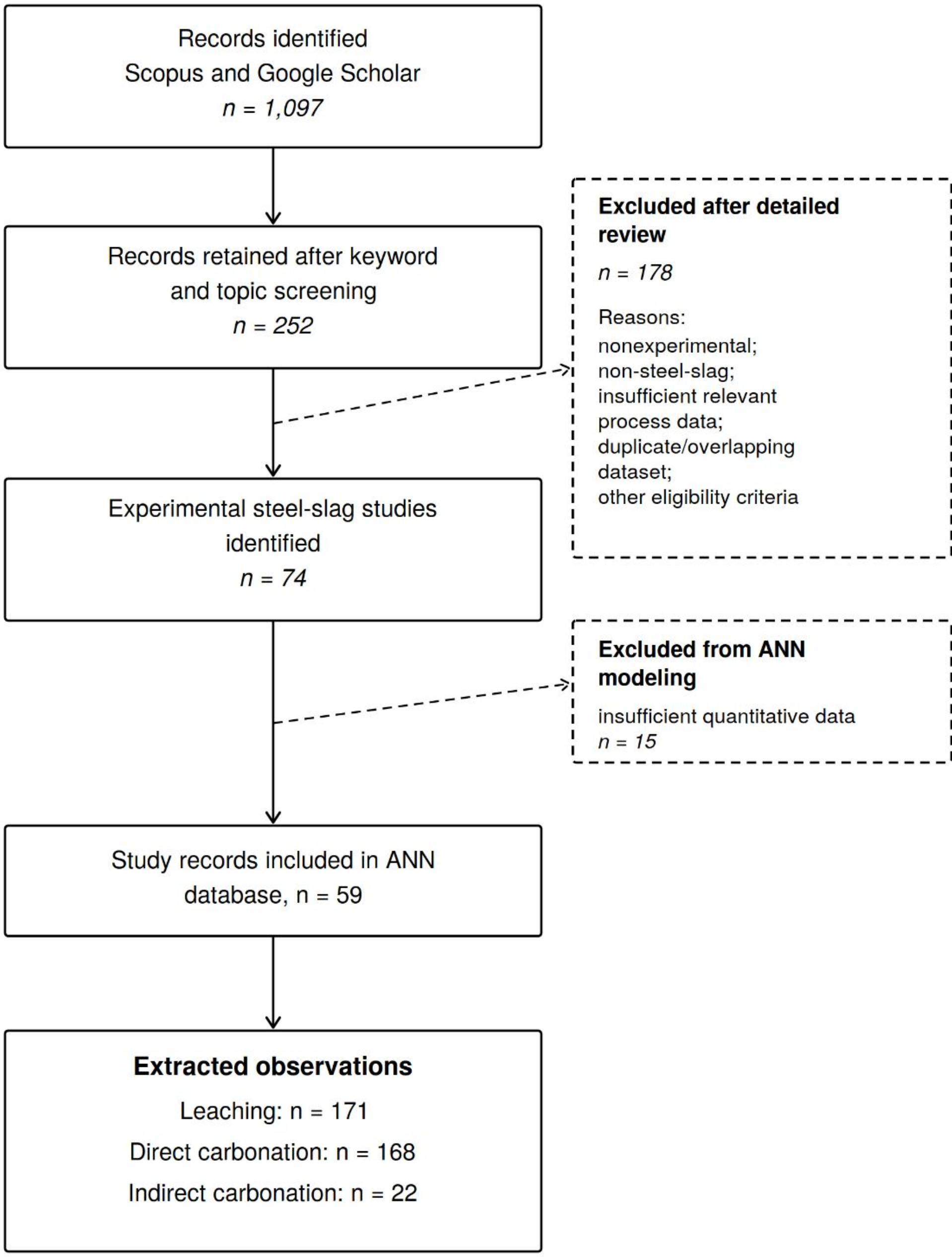

Figure S1. Literature search and study-selection flow diagram showing the screening of the initial literature pool and selection of study records used for ANN model development.

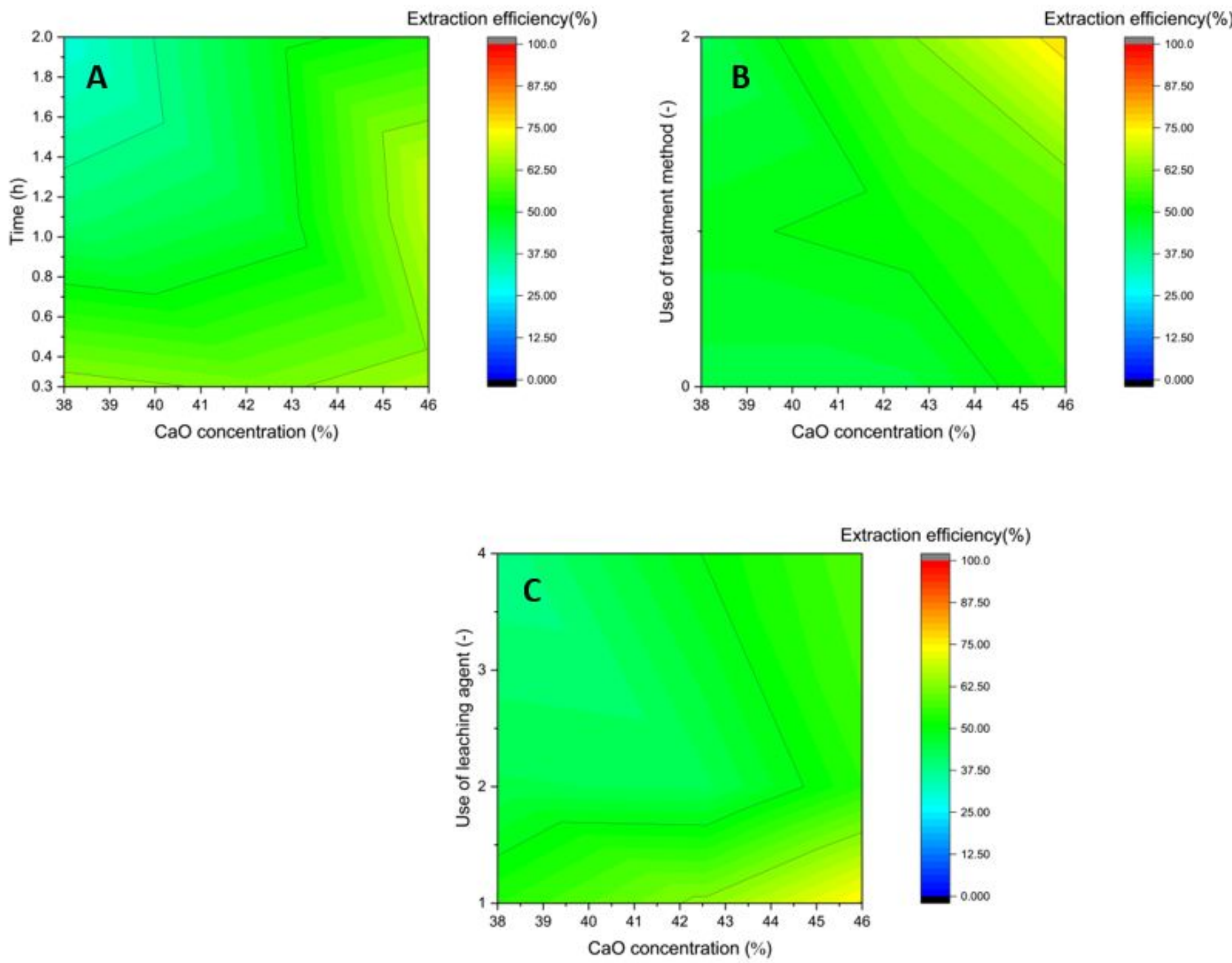

Figure S2. Contour plots of interaction between critical affecting factors on calcium extraction efficiency, created with 80% data used for modeling; (A) Time (Factor 5) vs. CaO concentration in feed (Factor 1), (B) use of treatment method (Factor 6) vs. CaO concentration in feed (Factor 1), (C) use of leaching agent (Factor 7) vs. CaO concentration in feed (Factor 1).

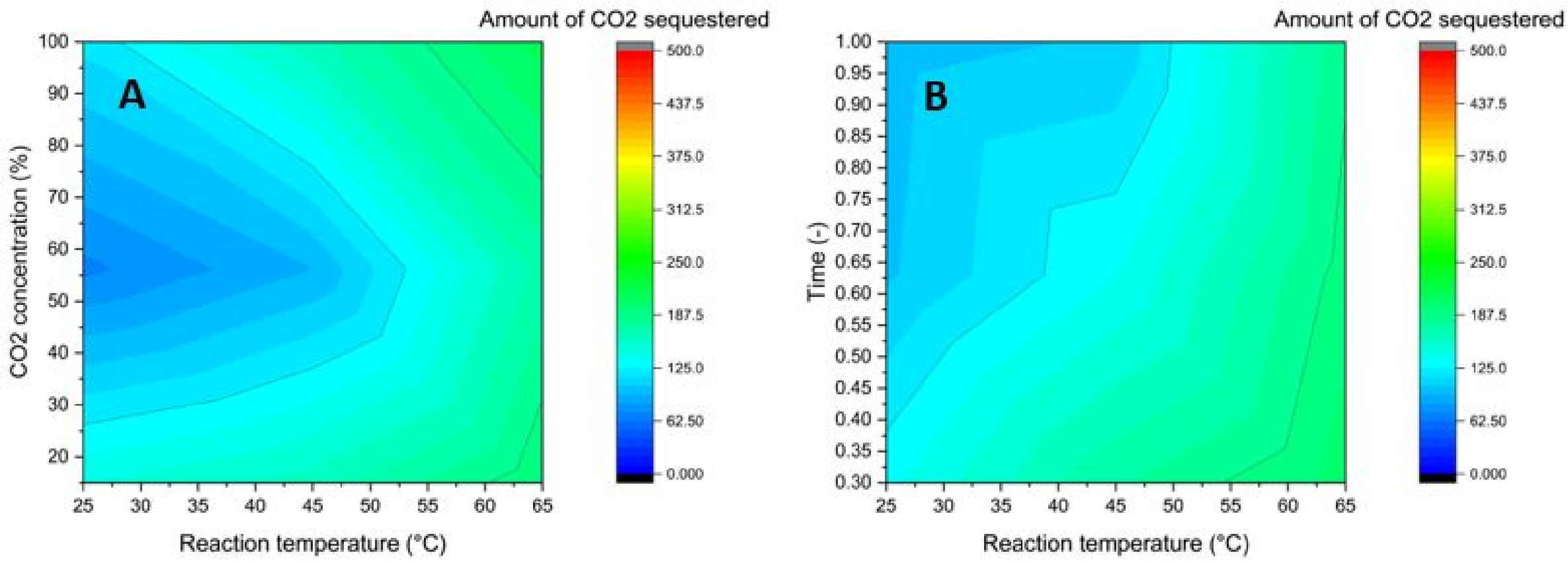

Figure S3. Contour plots of interaction between critical factors affecting indirect CO_2 mineralization capacity, created with 80% data used for modeling; (A) CO_2 concentration in feed (Factor 5) vs reaction temperature (Factor 2), (B) indirect carbonation time duration (Factor 6) vs. reaction temperature (Factor 2)

Table S1. Article library used for detailed analysis

Article Index	Used for extraction study	Used for carbonation study	Reference
1		✓*	Z. Wang et al., “High-efficiency CO2 sequestration through direct aqueous carbonation of carbide slag: determination of carbonation reaction and optimization of operation parameters,” Front. Environ. Sci. Eng., vol. 18, no. 1, p. 12, Jan. 2024, doi: 10.1007/s11783-024-1772-y.
2	✓	✓	Y. Zhang et al., “Performance and mechanisms of alkaline solid waste in CO2 mineralization and utilization,” Waste Management, vol. 175, pp. 62–72, Mar. 2024, doi: 10.1016/j.wasman.2023.12.047.
3		✓	Y.-R. Yi, Y. Lin, Y.-C. Du, S. Bai, Z. Ma, and Y. Chen, “Accelerated carbonation of ladle furnace slag and characterization of its mineral phase,” Construction and Building Materials, vol. 276, p. 122235, Mar. 2021, doi: 10.1016/j.conbuildmat.2020.122235.
4		✓	R. Baciocchi, G. Costa, A. Polettini, R. Pomi, A. Stramazzo, and D. Zingaretti, “Accelerated Carbonation of Steel Slags Using CO2 Diluted Sources: CO2 Uptakes and Energy Requirements,” Front. Energy Res., vol. 3, Jan. 2016, doi: 10.3389/fenrg.2015.00056.
5		✓	E.-E. Chang, S.-Y. Pan, Y.-H. Chen, C.-S. Tan, and P.-C. Chiang, “Accelerated carbonation of steelmaking slags in a high-gravity rotating packed bed,” Journal of Hazardous Materials, vol. 227–228, pp. 97–106, Aug. 2012, doi: 10.1016/j.jhazmat.2012.05.021.
6		✓	P. Librandi, G. Costa, A. C. B. D. Souza, S. Stendardo, A. S. Luna, and R. Baciocchi, “Carbonation of Steel Slag: Testing of the Wet Route in a Pilot-scale Reactor,” Energy Procedia, vol. 114, pp. 5381–5392, Jul. 2017, doi: 10.1016/j.egypro.2017.03.1674.
7		✓	A. Van Zomeren, S. R. Van Der Laan, H. B. A. Kobesen, W. J. J. Huijgen, and R. N. J. Comans, “Changes in mineralogical and leaching properties of converter steel slag resulting from accelerated carbonation at low CO2 pressure,” Waste Management, vol. 31, no. 11, pp. 2236–2244, Nov. 2011, doi: 10.1016/j.wasman.2011.05.022.
8		✓	T.-L. Chen, W. Jiang, A.-L. Shen, Y.-H. Chen, S.-Y. Pan, and P.-C. Chiang, “CO 2 Mineralization and Utilization Using Various Calcium-Containing

			Wastewater and Refining Slag via a High-Gravity Carbonation Process," Ind. Eng. Chem. Res., vol. 59, no. 15, pp. 7140–7150, Apr. 2020, doi: 10.1021/acs.iecr.9b05410.
9		✓*	D. Bonenfant et al., "CO 2 Sequestration Potential of Steel Slags at Ambient Pressure and Temperature," Ind. Eng. Chem. Res., vol. 47, no. 20, pp. 7610–7616, Oct. 2008, doi: 10.1021/ie701721j.
10	✓	✓	S. Lee, J.-W. Kim, S. Chae, J.-H. Bang, and S.-W. Lee, "CO2 sequestration technology through mineral carbonation: An extraction and carbonation of blast slag," Journal of CO2 Utilization, vol. 16, pp. 336–345, Dec. 2016, doi: 10.1016/j.jcou.2016.09.003.
11		✓	A. Polettini, R. Pomi, and A. Stramazzo, "CO2 sequestration through aqueous accelerated carbonation of BOF slag: A factorial study of parameters effects," Journal of Environmental Management, vol. 167, pp. 185–195, Feb. 2016, doi: 10.1016/j.jenvman.2015.11.042.
12	✓	✓	S. Kodama, T. Nishimoto, N. Yamamoto, K. Yogo, and K. Yamada, "Development of a new pH-swing CO2 mineralization process with a recyclable reaction solution," Energy, vol. 33, no. 5, pp. 776–784, May 2008, doi: 10.1016/j.energy.2008.01.005.
13		✓	T. D. Rushendra Revathy, K. Palanivelu, and A. Ramachandran, "Direct mineral carbonation of steelmaking slag for CO2 sequestration at room temperature," Environ Sci Pollut Res, vol. 23, no. 8, pp. 7349–7359, Apr. 2016, doi: 10.1007/s11356-015-5893-5.
14	✓		M. Dri, A. Sanna, and M. M. Maroto-Valer, "Dissolution of steel slag and recycled concrete aggregate in ammonium bisulphate for CO2 mineral carbonation," Fuel Processing Technology, vol. 113, pp. 114–122, Sep. 2013, doi: 10.1016/j.fuproc.2013.03.034.
15	✓*		S. Teir, S. Eloneva, C.-J. Fogelholm, and R. Zevenhoven, "Dissolution of steelmaking slags in acetic acid for precipitated calcium carbonate production," Energy, vol. 32, no. 4, pp. 528–539, Apr. 2007, doi: 10.1016/j.energy.2006.06.023.
16	✓	✓	S. Eloneva, S. Teir, J. Salminen, C.-J. Fogelholm, and R. Zevenhoven, "Fixation of CO2 by carbonating calcium derived from blast furnace slag," Energy, vol. 33, no. 9, pp. 1461–1467, Sep. 2008, doi: 10.1016/j.energy.2008.05.003.

17		✓	F. Zhu et al., “Experimental Investigation and Mechanism Analysis of Direct Aqueous Mineral Carbonation Using Steel Slag,” Sustainability, vol. 16, no. 1, p. 81, Dec. 2023, doi: 10.3390/su16010081.
18	✓	✓	Y. Sun, M.-S. Yao, J.-P. Zhang, and G. Yang, “Indirect CO2 mineral sequestration by steelmaking slag with NH4Cl as leaching solution,” Chemical Engineering Journal, vol. 173, no. 2, pp. 437–445, Sep. 2011, doi: 10.1016/j.cej.2011.08.002.
19		✓	M. Mun and H. Cho, “Mineral Carbonation for Carbon Sequestration with Industrial Waste,” Energy Procedia, vol. 37, pp. 6999–7005, 2013, doi: 10.1016/j.egypro.2013.06.633.
20	✓		S. Eloneva, A. Said, C.-J. Fogelholm, and R. Zevenhoven, “Preliminary assessment of a method utilizing carbon dioxide and steelmaking slags to produce precipitated calcium carbonate,” Applied Energy, vol. 90, no. 1, pp. 329–334, Feb. 2012, doi: 10.1016/j.apenergy.2011.05.045.
21	✓		A. Said, H.-P. Mattila, M. Järvinen, and R. Zevenhoven, “Production of precipitated calcium carbonate (PCC) from steelmaking slag for fixation of CO2,” Applied Energy, vol. 112, pp. 765–771, Dec. 2013, doi: 10.1016/j.apenergy.2012.12.042.
22	✓		Z. Tong, G. Ma, D. Zhou, G. Yang, and C. Peng, “The Indirect Mineral Carbonation of Electric Arc Furnace Slag Under Microwave Irradiation,” Sci Rep, vol. 9, no. 1, p. 7676, May 2019, doi: 10.1038/s41598-019-44162-x.
23		✓	N. L. Ukwattage, P. G. Ranjith, and X. Li, “Steel-making slag for mineral sequestration of carbon dioxide by accelerated carbonation,” Measurement, vol. 97, pp. 15–22, Feb. 2017, doi: 10.1016/j.measurement.2016.10.057.
24		✓	R. Baciocchi, G. Costa, M. Di Gianfilippo, A. Polettini, R. Pomi, A. Stramazzo, “Thin-film versus slurry-phase carbonation of steel slag: CO2 uptake and effects on mineralogy,” Journal of Hazardous Materials, vol. 283, pp. 302–313, Feb. 2015, doi: 10.1016/j.jhazmat.2014.09.016.
25		✓	M. Mun and H. Cho, “Mineral Carbonation for Carbon Sequestration with Industrial Waste,” Energy Procedia, vol. 37, pp. 6999–7005, 2013, doi: 10.1016/j.egypro.2013.06.633
26	✓*		Frédéric J. Doucet and F. J. Doucet, “Effective CO2-specific sequestration capacity of steel slags and

			variability in their leaching behaviour in view of industrial mineral carbonation," Minerals Engineering, vol. 23, no. 3, pp. 262–269, Feb. 2010, doi: 10.1016/j.mineng.2009.09.006.
27		✓	J. Kim and G. Azimi, "The CO2 sequestration by supercritical carbonation of electric arc furnace slag," Journal of CO2 Utilization, vol. 52, p. 101667, Oct. 2021, doi: 10.1016/j.jcou.2021.101667.
28		✓*	M. Uibu, R. Kuusik, L. Andreas, and K. Kirsimäe, "The CO2 -binding by Ca-Mg-silicates in direct aqueous carbonation of oil shale ash and steel slag," Energy Procedia, vol. 4, pp. 925–932, 2011, doi: 10.1016/j.egypro.2011.01.138.
29		✓	L. Li et al., "Research on integrated CO2 absorption-mineralization and regeneration of absorbent process," Energy, vol. 222, p. 120010, May 2021, doi: 10.1016/j.energy.2021.120010.
30		✓	Tu, M.; Zhao, H.; Lei, Z.; Wang, L.; Chen, D.; Yu, H.; Qi, T. Aqueous Carbonation of Steel Slag: A Kinetics Study. ISIJ International 2015, 55 (11), 2509–2514. https://doi.org/10.2355/isijinternational.ISIJINT-2015-142.
31		✓	Ma, M.; Mehdizadeh, H.; Guo, M.-Z.; Ling, T.-C. Effect of Direct Carbonation Routes of Basic Oxygen Furnace Slag (BOFS) on Strength and Hydration of Blended Cement Paste. Construction and Building Materials 2021, 304, 124628. https://doi.org/10.1016/j.conbuildmat.2021.124628.
32		✓	Pan, S.-Y.; Liu, H.-L.; Chang, E.-E.; Kim, H.; Chen, Y.-H.; Chiang, P.-C. Multiple Model Approach to Evaluation of Accelerated Carbonation for Steelmaking Slag in a Slurry Reactor. Chemosphere 2016, 154, 63–71. https://doi.org/10.1016/j.chemosphere.2016.03.093.
33		✓	Ghacham, A. B.; Pasquier, L.-C.; Cecchi, E.; Blais, J.-F.; Mercier, G. CO2 Sequestration by Mineral Carbonation of Steel Slags under Ambient Temperature: Parameters Influence, and Optimization. Environ Sci Pollut Res 2016, 23 (17), 17635–17646. https://doi.org/10.1007/s11356-016-6926-4.
34		✓	Omale, S. O.; Choong, T. S. Y.; Abdullah, L. C.; Siajam, S. I.; Yip, M. W. Utilization of Malaysia EAF Slags for Effective Application in Direct Aqueous Sequestration of Carbon Dioxide under Ambient Temperature. Heliyon 2019, 5 (10), e02602. https://doi.org/10.1016/j.heliyon.2019.e02602.

35	✓		Arshe Said et al., “Enhancement of calcium dissolution from steel slag by ultrasound,” Chemical Engineering and Processing, vol. 89, pp. 1–8, Mar. 2015, doi: 10.1016/j.cep.2014.12.008.
36		✓	E. E. Chang et al., “Carbonation of basic oxygen furnace slag with metalworking wastewater in a slurry reactor,” International Journal of Greenhouse Gas Control, vol. 12, pp. 382–389, Jan. 2013, doi: 10.1016/j.ijggc.2012.11.026.
37		✓*	E. E. Chang et al., “Performance evaluation for carbonation of steel-making slags in a slurry reactor,” Journal of Hazardous Materials, vol. 186, no. 1, pp. 558–564, Feb. 2011, doi: 10.1016/j.jhazmat.2010.11.038.
38		✓	E. E. Chang et al., “CO2 sequestration by carbonation of steelmaking slags in an autoclave reactor,” Journal of Hazardous Materials, vol. 195, pp. 107–114, Nov. 2011, doi: 10.1016/j.jhazmat.2011.08.006.
39		✓	K.-W. Chen, S.-Y. Pan, C.-T. Chen, Y.-H. Chen, and P.-C. Chiang, “High-gravity carbonation of basic oxygen furnace slag for CO2 fixation and utilization in blended cement,” Journal of Cleaner Production, vol. 124, pp. 350–360, Jun. 2016, doi: 10.1016/j.jclepro.2016.02.072.
40	✓	✓	H.-P. Mattila, I. Grigaliūnaitė, and R. Zevenhoven, “Chemical kinetics modeling and process parameter sensitivity for precipitated calcium carbonate production from steelmaking slags,” Chemical Engineering Journal, vol. 192, pp. 77–89, Jun. 2012, doi: 10.1016/j.cej.2012.03.068.
41		✓*	C. F. Matus, S. R. Stopić, and B. G. Friedrich, “Carbonation of minerals and slags under high pressure in an autoclave,” Vojnotehnički glasnik, vol. 69, no. 2, pp. 486–498, 2021, doi: 10.5937/vojtehg69-30203.
42	✓		Ragipani, S. Bhattacharya, and A. K. Suresh, “Kinetics of steel slag dissolution: from experiments to modelling,” Proceedings of the Royal Society A: Mathematical, Physical and Engineering Sciences, vol. 475, no. 2224, Apr. 2019.
43		✓	Zhenhao Wang, Chuanwen Zhao, Pu Huang, Yuxuan Zhang, Jian Sun,Modeling and response surface methodology optimization of reaction parameters for aqueous mineral carbonation by steel slag,Carbon Capture Science & Technology,Volume 12,2024,100229,ISSN 2772-6568,https://doi.org/10.1016/j.ccst.2024.100229.

44		✓*	Nannan Zhang, Gao Deng, Wenyu Liao, Hongyan Ma, Chuanlin Hu,Aqueous carbonation of steel slags: A comparative study on mechanisms,Cement and Concrete Composites,2024,105838,ISSN 0958-9465,https://doi.org/10.1016/j.cemconcomp.2024.105838.
45	✓	✓	Lincheng Liu, Xiaohui Fan, Min Gan, Jiaoyang Wei, Zitan Gao, Zengqing Sun, Zhiyun Ji, Yufeng Wu, Jinhua Li,Microwave-enhanced selective leaching calcium from steelmaking slag to fix CO2 and produce high value-added CaCO3,Separation and Purification Technology,Volume 330, Part B,2024,125395,ISSN 1383-5866,https://doi.org/10.1016/j.seppur.2023.125395.
46	✓*		Xiaohui Mei, Qing Zhao, Yi Min, Chengjun Liu, Peiyang Shi, Henrik Saxén, Ron Zevenhoven,Dissolution behavior of steelmaking slag for Ca extraction toward CO2 sequestration,Journal of Environmental Chemical Engineering,Volume 11, Issue 3,2023,110043,ISSN 2213-3437,https://doi.org/10.1016/j.jece.2023.110043.
47		✓*	Kamal Elyasi Gomari, Sina Rezaei Gomari, David Hughes, Tariq Ahmed,Exploring the potential of steel slag waste for carbon sequestration through mineral carbonation: A comparative study of blast-furnace slag and ladle slag,Journal of Environmental Management,Volume 351,2024,119835,ISSN 0301-4797,https://doi.org/10.1016/j.jenvman.2023.119835.
48		✓	Zijian Li, Jie Chen, Zongze Lv, Yichao Tong, Jingyu Ran, Changlei Qin,Evaluation on direct aqueous carbonation of industrial/mining solid wastes for CO2 mineralization,Journal of Industrial and Engineering Chemistry,Volume 122,2023,Pages 359-365,ISSN 1226-086X,https://doi.org/10.1016/j.jiec.2023.02.036.
49	✓		Yarong Li, Hongqiang Liao, Chao Li, Zhibin Ma, Yanxia Guo,Dissolution characteristics of steel slag in aqueous solution and preparation of high purity CaCO3, Process Safety and Environmental Protection, Volume 198,2025, 107183,
50	✓	✓	Yarong Li, Hongqiang Liao, Yanxia Guo,Aqueous carbonation of steel slag and preparation of calcium carbon: A new strategy for the utilization of steel slag, Journal of Environmental Chemical Engineering, Volume 13, Issue 2, 2025, 115951,
51	✓		Liu, F.; Zhao, L.; Zhu, C.; Li, G. K. Dissolution Kinetics of Steel Slag in Ammonium Chloride Solutions

			for Carbon Mineralization: An Experimental Study and Implications for Carbon Capture and Sequestration. Applied Geochemistry 2026, 205, 106883. https://doi.org/10.1016/j.apgeochem.2026.106883.
52	✓		Dongfeng He, Liu Yang, Yinbo Luo, Guoping Liu, Zhanjun Wu, Synergistic calcium leaching and iron enrichment by indirect carbonation of thermally modified steel slag, Construction and Building Materials, Volume 411, 2024, 134249, ISSN 0950-0618, https://doi.org/10.1016/j.conbuildmat.2023.134249.
53	✓		Can Cheng, Wenjun Huang, Haomiao Xu, Zhisong Liu, Xianwei Li, Hongzhi Shi, Yongmei Yu, Zan Qu, Naiqiang Yan, CO2 sequestration and CaCO3 recovery with steel slag by a novel two-step leaching and carbonation method, Science of The Total Environment, Volume 891, 2023, 164203, ISSN 0048-9697, https://doi.org/10.1016/j.scitotenv.2023.164203.
54		✓	Zhengqi Zheng, Xingyang He, Huahui Qi, Ying Su, Hongbo Tan, Yingbin Wang, Jin Yang, Yubo Li, A new method to improve the carbon mineralization efficiency of steel slag in aqueous condition: The effect of mechanical exfoliation, Journal of Building Engineering, Volume 106, 2025, 112473, ISSN 2352-7102, https://doi.org/10.1016/j.jobe.2025.112473.
55	✓		Luo, Y., He, D. Research on Indirect Carbonation of Two-step Leaching for the Purpose of Utilizing the Alkalinity of Steel Slag. J. Sustain. Metall. 7, 947–963 (2021). https://doi.org/10.1007/s40831-021-00384-w
56		✓	Huijgen WJJ, Witkamp G-J, Comans RNJ. Mineral CO2 sequestration by steel slag carbonation. Environ Sci Technol. 2005;39(24). doi:10.1021/es050795f
57	✓	✓	Arshe Said, Timo Laukkanen, Mika Järvinen, Pilot-scale experimental work on carbon dioxide sequestration using steelmaking slag, Applied Energy, Volume 177, 2016, Pages 602-611, ISSN 0306-2619, https://doi.org/10.1016/j.apenergy.2016.05.136
58	✓	✓	Radfarnia H., Staneva K., Shafeen A., Zanganeh K., Patarachao B., Vasquez Huertas S., Zborowski A., Kung J., Dashtban Kenari S. L., Mosadeghsedghi S., Volchek K, Experimental Study on Indirect CO_2 Mineralization of Industrial Solid Wastes: Electric Arc Furnace (EAF) Slag and Nickel Mine Tailings. ACS Omega 2025, *10* (36), 41571-41585. https://doi.org/10.1021/acsomega.5c05128

*multiple types of slag used

Table S2. Results for ANOVA Statistical Analysis with 100% of extraction efficiency data*

Factor	SS**	D.f.***	F-value	p-value
Factor1	409960.5	2	612.46	0.00
Factor6	242239.7	2	361.90	0.00
Factor3	224106.5	2	334.81	0.00
Factor2	132673	2	198.21	0.00
Factor5	120289.6	2	179.71	0.00
Factor2:Factor5	165134.6	4	123.35	0.00
Factor3:Factor4	164351.4	4	122.77	0.00
Factor1:Factor4	123291.7	4	92.10	0.00
Factor1:Factor5	86997.45	4	64.99	0.00
Factor1:Factor2	86234.07	4	64.41	0.00
Factor7	38216.14	2	57.09	0.00
Factor1:Factor6	72871.25	4	54.43	0.00
Factor1:Factor3	66531.37	4	49.70	0.00
Factor2:Factor3	51083.81	4	38.16	0.00
Factor1:Factor2:Factor5	98962.96	8	36.96	0.00
Factor2:Factor4	46700.46	4	34.88	0.00
Factor1:Factor2:Factor6	80899.78	8	30.22	0.00
Factor2:Factor7	38509.37	4	28.77	0.00
Factor4	19017.77	2	28.41	0.00
Factor1:Factor3:Factor5	72182.62	8	26.96	0.00
Factor5:Factor7	35390.54	4	26.44	0.00
Factor1:Factor2:Factor3	61015.76	8	22.79	0.00
Factor1:Factor2:Factor7	59015.59	8	22.04	0.00
Factor2:Factor5:Factor6	51998.79	8	19.42	0.00
Factor5:Factor6	24808.63	4	18.53	0.00
Factor1:Factor2:Factor4	47445.26	8	17.72	0.00
Factor2:Factor3:Factor4	42945.56	8	16.04	0.00
Factor1:Factor3:Factor7	42106.08	8	15.73	0.00
Factor3:Factor5	19531.24	4	14.59	0.00
Factor1:Factor4:Factor5	39026.66	8	14.58	0.00
Factor1:Factor3:Factor4	38922.77	8	14.54	0.00
Factor2:Factor3:Factor5	35267.92	8	13.17	0.00
Factor1:Factor5:Factor7	34930.61	8	13.05	0.00
Factor6:Factor7	17121.03	4	12.79	0.00
Factor3:Factor4:Factor5	31961.94	8	11.94	0.00
Factor1:Factor5:Factor6	29785.44	8	11.12	0.00
Factor1:Factor3:Factor4:Factor5	51900.29	16	9.69	0.00
Factor1:Factor2:Factor3:Factor5	51559.8	16	9.63	0.00

Factor4:Factor7	12599.98	4	9.41	0.00
Factor2:Factor3:Factor7	24121.04	8	9.01	0.00
Factor1:Factor2:Factor3:Factor4	47648.62	16	8.90	0.00
Factor1:Factor2:Factor5:Factor7	43776.22	16	8.17	0.00
Factor2:Factor4:Factor5	20054.63	8	7.49	0.00
Factor2:Factor4:Factor6	19650.23	8	7.34	0.00
Factor3:Factor4:Factor7	19433.21	8	7.26	0.00
Factor1:Factor3:Factor6	17433.26	8	6.51	0.00
Factor1:Factor4:Factor5:Factor6	33779.64	16	6.31	0.00
Factor1:Factor4:Factor6	16673.72	8	6.23	0.00
Factor3:Factor5:Factor7	16157.15	8	6.03	0.00
Factor1:Factor2:Factor5:Factor6	30006.93	16	5.60	0.00
Factor4:Factor6	7466.771	4	5.58	0.00
Factor3:Factor4:Factor6	14862.21	8	5.55	0.00
Factor1:Factor2:Factor3:Factor6	29451.64	16	5.50	0.00
Factor2:Factor4:Factor7	14173.63	8	5.29	0.00
Factor1:Factor4:Factor7	13945.28	8	5.21	0.00
Factor1:Factor2:Factor3:Factor7	27319.24	16	5.10	0.00
Factor1:Factor3:Factor4:Factor6	26013.89	16	4.86	0.00
Factor3:Factor4:Factor5:Factor7	25810	16	4.819877	0.00
Factor1:Factor7	6405.879	4	4.79	0.00
Factor1:Factor5:Factor6:Factor7	25550.53	16	4.77	0.00
Factor2:Factor3:Factor4:Factor7	25200.23	16	4.71	0.00
Factor2:Factor3:Factor4:Factor5	25155.85	16	4.70	0.00
Factor2:Factor5:Factor7	12491.59	8	4.67	0.00
Factor1:Factor6:Factor7	12048.02	8	4.50	0.00
Factor1:Factor3:Factor4:Factor7	23550.85	16	4.40	0.00
Factor3:Factor5:Factor6	11674.11	8	4.36	0.00
Factor1:Factor2:Factor4:Factor7	21823.26	16	4.08	0.00
Factor1:Factor4:Factor6:Factor7	20870.87	16	3.90	0.00
Factor1:Factor2:Factor3:Factor5:Factor6	41338.75	32	3.859893	0.00
Factor1:Factor2:Factor6:Factor7	19728.55	16	3.68	0.00
Factor2:Factor3:Factor5:Factor6	19027.93	16	3.55	0.00
Factor2:Factor4:Factor5:Factor7	18296.42	16	3.416757	0.00
Factor2:Factor6:Factor7	8850.385	8	3.31	0.00
Factor3:Factor7	4419.771	4	3.30	0.01
Factor1:Factor2:Factor4:Factor5:Factor7	34820.89	32	3.251306	0.00
Factor5:Factor6:Factor7	8402.802	8	3.14	0.00
Factor2:Factor4:Factor5:Factor6	16090.96	16	3.0049	0.00
Factor1:Factor2:Factor5:Factor6:Factor7	31334.09	32	2.925735	0.00
Factor1:Factor2:Factor3:Factor4:Factor5	30564.66	32	2.853892	0.00
Factor2:Factor3:Factor5:Factor7	15262.96	16	2.85	0.00
Factor1:Factor2:Factor3:Factor6:Factor7	29991.94	32	2.800416	0.00

Factor3:Factor5:Factor6:Factor7	14540.48	16	2.715355	0.00
Factor2:Factor3:Factor4:Factor5:Factor7	27968.31	32	2.611465	0.00
Factor1:Factor3:Factor6:Factor7	13534.16	16	2.53	0.00
Factor3:Factor4:Factor5:Factor6	13258.64	16	2.475978	0.00
Factor2:Factor3:Factor6	6370.632	8	2.38	0.02
Factor4:Factor5:Factor6	6267.291	8	2.34	0.02
Factor2:Factor5:Factor6:Factor7	12184.39	16	2.275368	0.01
Factor1:Factor3:Factor5:Factor6:Factor7	22679.06	32	2.117595	0.00
Factor1:Factor2:Factor4:Factor5	11272.27	16	2.11	0.01
Factor1:Factor2:Factor4:Factor6	11186.19	16	2.09	0.01
Factor1:Factor2:Factor3:Factor5:Factor6:Factor7	44265.32	64	2.066577	0.00
Factor1:Factor3:Factor5:Factor6	10794.41	16	2.02	0.02
Factor4:Factor6:Factor7	5190.987	8	1.94	0.06
Factor2:Factor3:Factor6:Factor7	10072.13	16	1.880915	0.03
Factor1:Factor4:Factor5:Factor7	10034.71	16	1.87	0.03
Factor1:Factor3:Factor5:Factor7	10031.16	16	1.87	0.03
Factor1:Factor2:Factor3:Factor5:Factor7	19659.82	32	1.835682	0.01
Factor2:Factor3:Factor4:Factor6	9740.424	16	1.82	0.04
Factor1:Factor2:Factor4:Factor5:Factor6	17559.31	32	1.639553	0.03
Factor2:Factor3:Factor4:Factor6:Factor7	17342.44	32	1.619303	0.03
Factor1:Factor2:Factor3:Factor4:Factor5:Factor6	34550.13	64	1.613012	0.01
Factor1:Factor2:Factor3:Factor4:Factor6:Factor7	33998.59	64	1.587263	0.01
Factor4:Factor5	2123.525	4	1.59	0.18
Factor2:Factor3:Factor4:Factor5:Factor6:Factor7	33965.01	64	1.585695	0.01
Factor2:Factor4:Factor6:Factor7	8465.718	16	1.580927	0.08
Factor1:Factor3:Factor4:Factor5:Factor6	16651.95	32	1.554831	0.04

For compactness, only terms with nominal $p < 0.05$ are displayed. These nominal p-values are descriptive screening quantities for the ANN-generated surrogate response surface and are not interpreted as evidence of independent experimental statistical significance. **SS: sum of squares; D.f.: degrees of freedom.

Table S3. Results for ANOVA statistical analysis with 80% of extraction efficiency data*

Factor	SS**	D.f.***	F-value	p-value
Factor6:Factor7	440016.1	4	542.7425	0.00
Factor5	169658.1	2	418.5333	0.00
Factor1	160261.1	2	395.3516	0.00
Factor7	135811.6	2	335.0366	0.00
Factor6	88101.59	2	217.3397	0.00
Factor1:Factor4	175182	4	216.0801	0.00
Factor3:Factor7	157517.2	4	194.2912	0.00
Factor2:Factor5	143593.4	4	177.1169	0.00
Factor1:Factor5	93770.37	4	115.6621	0.00
Factor3:Factor6	68442.91	4	84.42164	0.00
Factor1:Factor6	67500.41	4	83.2591	0.00
Factor3:Factor6:Factor7	130034.6	8	80.1963	0.00
Factor2:Factor6	64081.34	4	79.04182	0.00
Factor5:Factor6	58817.4	4	72.54896	0.00
Factor4:Factor7	52453.93	4	64.69986	0.00
Factor1:Factor6:Factor7	80771.94	8	49.81451	0.00
Factor4	19885.83	2	49.05678	0.00
Factor1:Factor2	38555.53	4	47.55674	0.00
Factor2	19152.83	2	47.24851	0.00
Factor2:Factor6:Factor7	73879.4	8	45.56367	0.00
Factor1:Factor5:Factor7	66213.45	8	40.83584	0.00
Factor3:Factor4	28599.86	4	35.2768	0.00
Factor5:Factor7	28369.42	4	34.99256	0.00
Factor3:Factor5	28100.85	4	34.6613	0.00
Factor2:Factor4	27603.53	4	34.04787	0.00
Factor5:Factor6:Factor7	55091.02	8	33.9763	0.00
Factor1:Factor5:Factor6	46206.18	8	28.49675	0.00
Factor4:Factor6	22732.83	4	28.04006	0.00
Factor1:Factor3	18486.95	4	22.80293	0.00
Factor4:Factor6:Factor7	35200.34	8	21.70912	0.00
Factor1:Factor4:Factor7	31665.07	8	19.52881	0.00
Factor1:Factor7	15456.42	4	19.06488	0.00
Factor2:Factor3	14711.76	4	18.14638	0.00
Factor2:Factor3:Factor5	29296.6	8	18.0681	0.00
Factor3:Factor4:Factor5	29001.85	8	17.88632	0.00
Factor1:Factor3:Factor6	28699.61	8	17.69992	0.00
Factor2:Factor5:Factor6	27164.61	8	16.75324	0.00
Factor2:Factor3:Factor4	26268.29	8	16.20045	0.00

Factor4:Factor5	12692.4	4	15.65558	0.00
Factor3:Factor5:Factor6:Factor7	49528.62	16	15.2729	0.00
Factor2:Factor3:Factor7	23693.1	8	14.61225	0.00
Factor3:Factor4:Factor7	23449.7	8	14.46214	0.00
Factor1:Factor2:Factor7	22975.52	8	14.1697	0.00
Factor1:Factor3:Factor5	21191.22	8	13.06927	0.00
Factor2:Factor4:Factor7	20338.34	8	12.54327	0.00
Factor2:Factor3:Factor6	20009.38	8	12.34039	0.00
Factor2:Factor3:Factor6:Factor7	39998.77	16	12.33423	0.00
Factor1:Factor4:Factor5:Factor7	35425.41	16	10.92396	0.00
Factor3:Factor5:Factor6	16957.24	8	10.45804	0.00
Factor1:Factor3:Factor6:Factor7	33275.2	16	10.26091	0.00
Factor2:Factor4:Factor5:Factor7	32234.61	16	9.940032	0.00
Factor2:Factor7	7862.487	4	9.698068	0.00
Factor1:Factor2:Factor6:Factor7	30423.67	16	9.381601	0.00
Factor1:Factor4:Factor5	14769.52	8	9.108809	0.00
Factor1:Factor4:Factor6	14736.26	8	9.088296	0.00
Factor2:Factor3:Factor5:Factor6:Factor7	57125.73	32	8.807793	0.00
Factor3:Factor4:Factor6	13202.7	8	8.142507	0.00
Factor2:Factor3:Factor5:Factor6	22142.19	16	6.827881	0.00
Factor1:Factor2:Factor3	10723.56	8	6.613544	0.00
Factor3:Factor5:Factor7	10504.95	8	6.478722	0.00
Factor1:Factor2:Factor4	10453.1	8	6.446744	0.00
Factor2:Factor3:Factor4:Factor7	19749.1	16	6.089934	0.00
Factor1:Factor3:Factor5:Factor7	19260.48	16	5.93926	0.00
Factor2:Factor4:Factor6:Factor7	17535.27	16	5.407268	0.00
Factor1:Factor3:Factor7	8346.32	8	5.147429	0.00
Factor2:Factor4:Factor6	8084.487	8	4.985948	0.00
Factor4:Factor5:Factor7	7980.337	8	4.921716	0.00
Factor1:Factor2:Factor5	7969.436	8	4.914993	0.00
Factor1:Factor4:Factor6:Factor7	15217.32	16	4.692492	0.00
Factor1:Factor3:Factor5:Factor6	14181.21	16	4.37299	0.00
Factor2:Factor5:Factor7	6805.794	8	4.19734	0.00
Factor1:Factor3:Factor4:Factor7	13418.65	16	4.137845	0.00
Factor2:Factor5:Factor6:Factor7	13136.38	16	4.050802	0.00
Factor4:Factor5:Factor6	6541.761	8	4.034502	0.00
Factor1:Factor2:Factor3:Factor6	12993.32	16	4.006687	0.00
Factor1:Factor3:Factor5:Factor6:Factor7	24685.14	32	3.806019	0.00
Factor1:Factor2:Factor6	6078.009	8	3.748493	0.00
Factor2:Factor3:Factor5:Factor7	11801.64	16	3.639213	0.00
Factor1:Factor5:Factor6:Factor7	11801.53	16	3.639181	0.00
Factor3:Factor4:Factor5:Factor6:Factor7	20450.07	32	3.153045	0.00
Factor3:Factor4:Factor6:Factor7	10178.17	16	3.138594	0.00

Factor4:Factor5:Factor6:Factor7	9717.294	16	2.996475	0.00
Factor3:Factor4:Factor5:Factor6	8751.613	16	2.698693	0.00
Factor2:Factor4:Factor5	4298.627	8	2.651094	0.01
Factor1:Factor2:Factor5:Factor7	8384.046	16	2.585348	0.00
Factor1:Factor4:Factor5:Factor6:Factor7	16300.66	32	2.513278	0.00
Factor1:Factor3:Factor4:Factor6	8139.774	16	2.510023	0.00
Factor2:Factor4:Factor5:Factor6:Factor7	15960.23	32	2.46079	0.00
Factor1:Factor3:Factor4	3754.38	8	2.31544	0.02
Factor1:Factor2:Factor5:Factor6:Factor7	14922.27	32	2.300754	0.00
Factor1:Factor2:Factor3:Factor5:Factor6	14865.98	32	2.292076	0.00
Factor1:Factor2:Factor3:Factor6:Factor7	14351.73	32	2.212787	0.00
Factor2:Factor4:Factor5:Factor6	6967.929	16	2.148667	0.01
Factor1:Factor3:Factor4:Factor5	6757.992	16	2.083929	0.01
Factor1:Factor2:Factor4:Factor6:Factor7	12950.36	32	1.996719	0.00
Factor1:Factor2:Factor3:Factor5:Factor6:Factor7	25884.81	64	1.995494	0.00
Factor1:Factor3:Factor4:Factor6:Factor7	12501.41	32	1.9275	0.01
Factor1:Factor2:Factor4:Factor7	6079.071	16	1.874574	0.03
Factor1:Factor2:Factor3:Factor4	6044.757	16	1.863993	0.03
Factor1:Factor2:Factor5:Factor6	6044.089	16	1.863787	0.03
Factor2:Factor3:Factor4:Factor5	5976.679	16	1.843	0.03
Factor1:Factor3:Factor4:Factor5:Factor6:Factor7	23233.59	64	1.791108	0.00

For compactness, only terms with nominal $p < 0.05$ are displayed. These nominal p-values are descriptive screening quantities for the ANN-generated surrogate response surface and are not interpreted as evidence of independent experimental statistical significance. **SS: sum of squares; D.f.: degrees of freedom.

Table S4: Results for ANOVA statistical analysis with 100% of CO_2 sequestration capacity data-direct carbonation*

Factor	SS*	D.f.***	F-value	p-value
Factor5	238513700	2	2500.30	0.00
Factor7	116522000	2	1221.48	0.00
Factor6	103741200	2	1087.50	0.00
Factor1:Factor5	99386850	4	520.93	0.00
Factor1	45083370	2	472.60	0.00
Factor7:Factor8	64199290	4	336.50	0.00
Factor5:Factor7:Factor8	126547200	8	331.6435	0.00
Factor1:Factor4	63064070	4	330.55	0.00
Factor1:Factor2	52415870	4	274.73	0.00
Factor2:Factor8	50157820	4	262.90	0.00
Factor1:Factor4:Factor5	100266200	8	262.77	0.00
Factor1:Factor4:Factor8	97571890	8	255.71	0.00
Factor4:Factor5	44227370	4	231.81	0.00
Factor1:Factor3	43390430	4	227.43	0.00
Factor2:Factor6	42630360	4	223.44	0.00
Factor4:Factor5:Factor8	71714760	8	187.9435	0.00
Factor5:Factor7	34620050	4	181.46	0.00
Factor5:Factor6	32348690	4	169.55	0.00
Factor2:Factor4:Factor7	62845320	8	164.70	0.00
Factor4	15369820	2	161.12	0.00
Factor2	14942160	2	156.64	0.00
Factor2:Factor4	28505740	4	149.41	0.00
Factor1:Factor2:Factor7	55670550	8	145.90	0.00
Factor5:Factor8	25771500	4	135.08	0.00
Factor1:Factor7:Factor8	50030660	8	131.12	0.00
Factor1:Factor5:Factor8	49961500	8	130.93	0.00
Factor3	12157960	2	127.45	0.00
Factor4:Factor7:Factor8	48120380	8	126.11	0.00
Factor1:Factor2:Factor6	44241110	8	115.94	0.00
Factor1:Factor7	21822340	4	114.38	0.00
Factor4:Factor7	20539790	4	107.6576	0.00
Factor1:Factor2:Factor8	40290780	8	105.59	0.00
Factor2:Factor5	17332260	4	90.85	0.00
Factor3:Factor8	17105910	4	89.66	0.00
Factor4:Factor5:Factor7	34052330	8	89.24	0.00
Factor1:Factor6	15257250	4	79.97	0.00
Factor8	7505957	2	78.68	0.00
Factor1:Factor4:Factor6	29874390	8	78.29	0.00
Factor4:Factor6	14563490	4	76.33	0.00

Factor4:Factor8	12596930	4	66.03	0.00
Factor1:Factor4:Factor5:Factor8	47604370	16	62.3786	0.00
Factor2:Factor5:Factor6:Factor7	45681490	16	59.85894	0.00
Factor1:Factor4:Factor7:Factor8	45448690	16	59.5539	0.00
Factor6:Factor7:Factor8	22422610	8	58.76313	0.00
Factor5:Factor6:Factor8	21653910	8	56.75	0.00
Factor1:Factor8	10652820	4	55.84	0.00
Factor1:Factor3:Factor4	19785980	8	51.85	0.00
Factor3:Factor4	8876005	4	46.52	0.00
Factor2:Factor5:Factor7	17669630	8	46.30695	0.00
Factor2:Factor3:Factor5	16936130	8	44.38	0.00
Factor5:Factor6:Factor7:Factor8	33436050	16	43.81308	0.00
Factor2:Factor6:Factor7	16628840	8	43.57936	0.00
Factor1:Factor2:Factor5:Factor8	29914890	16	39.19911	0.00
Factor4:Factor5:Factor7:Factor8	29542720	16	38.71144	0.00
Factor2:Factor5:Factor6	13134640	8	34.42	0.00
Factor1:Factor4:Factor5:Factor7:Factor8	48936890	32	32.06233	0.00
Factor1:Factor3:Factor8	12071110	8	31.63	0.00
Factor1:Factor2:Factor5	11769540	8	30.84	0.00
Factor1:Factor2:Factor4	11649850	8	30.53	0.00
Factor2:Factor4:Factor5:Factor8	22505040	16	29.48958	0.00
Factor1:Factor4:Factor7	11182460	8	29.30598	0.00
Factor3:Factor4:Factor5	9874110	8	25.87717	0.00
Factor2:Factor6:Factor8	9712075	8	25.45	0.00
Factor1:Factor2:Factor6:Factor7	17991580	16	23.57535	0.00
Factor1:Factor4:Factor6:Factor8	17331990	16	22.71105	0.00
Factor2:Factor3:Factor8	8632754	8	22.62	0.00
Factor2:Factor3:Factor4	8138268	8	21.33	0.00
Factor3:Factor4:Factor7	8033011	8	21.05	0.00
Factor1:Factor2:Factor6:Factor8	15197360	16	19.91	0.00
Factor4:Factor6:Factor8	7380460	8	19.34204	0.00
Factor1:Factor5:Factor7	7202203	8	18.87	0.00
Factor3:Factor4:Factor8	7011654	8	18.38	0.00
Factor5:Factor6:Factor7	6915376	8	18.12319	0.00
Factor3:Factor4:Factor5:Factor8	12947470	16	16.96577	0.00
Factor2:Factor4:Factor8	6260493	8	16.41	0.00
Factor1:Factor3:Factor7	6134987	8	16.08	0.00
Factor1:Factor3:Factor4:Factor6	12086850	16	15.83806	0.00
Factor3:Factor6:Factor7	5966719	8	15.63704	0.00
Factor3:Factor4:Factor6:Factor8	11802850	16	15.46592	0.00
Factor3:Factor4:Factor5:Factor7	11790270	16	15.44943	0.00
Factor1:Factor2:Factor4:Factor8	11727230	16	15.36683	0.00
Factor1:Factor5:Factor7:Factor8	10848660	16	14.21559	0.00
Factor3:Factor7	2641120	4	13.84	0.00
Factor1:Factor3:Factor5:Factor8	10254470	16	13.44	0.00

Factor1:Factor4:Factor5:Factor6	10209460	16	13.37801	0.00
Factor3:Factor5:Factor6:Factor8	9885094	16	12.95298	0.00
Factor1:Factor6:Factor8	4915870	8	12.88	0.00
Factor1:Factor2:Factor3:Factor7	9713352	16	12.73	0.00
Factor1:Factor2:Factor5:Factor7	9354140	16	12.25724	0.00
Factor2:Factor4:Factor6:Factor8	9347963	16	12.24915	0.00
Factor2:Factor5:Factor8	4509985	8	11.82	0.00
Factor2:Factor5:Factor7:Factor8	8932908	16	11.70528	0.00
Factor2:Factor3:Factor4:Factor7	8260317	16	10.82394	0.00
Factor4:Factor5:Factor6:Factor8	8244937	16	10.80379	0.00
Factor1:Factor3:Factor6	4122265	8	10.80	0.00
Factor1:Factor2:Factor4:Factor5:Factor8	16405250	32	10.74835	0.00
Factor3:Factor5:Factor8	4037221	8	10.58038	0.00
Factor1:Factor2:Factor4:Factor7	7892747	16	10.3423	0.00
Factor2:Factor3:Factor4:Factor5	7780146	16	10.19475	0.00
Factor3:Factor7:Factor8	3882718	8	10.17547	0.00

For compactness, only terms with nominal $p < 0.05$ are displayed. These nominal p-values are descriptive screening quantities for the ANN-generated surrogate response surface and are not interpreted as evidence of independent experimental statistical significance. **SS: sum of squares; D.f.: degrees of freedom.

Table S5. Results for ANOVA statistical analysis with 80% of CO_2 sequestration capacity data-direct carbonation

Factor	SS*	D.f.***	F-value	p-value
Factor5	1.541E+09	2	2780672	0.00
Factor6	465391700	2	839724.6	0.00
Factor4:Factor7	317343000	4	286297.2	0.00
Factor5:Factor6	260541400	4	235052.5	0.00
Factor1:Factor7	253371500	4	228584.1	0.00
Factor7	102019100	2	184077.1	0.00
Factor5:Factor6:Factor7	335057900	8	151139.5	0.00
Factor8	73208100	2	132092.3	0.00
Factor6:Factor7	84066840	4	75842.56	0.00
Factor5:Factor8	68016350	4	61362.3	0.00
Factor5:Factor7	50753980	4	45788.71	0.00
Factor1:Factor4	48025710	4	43327.34	0.00
Factor1	23114020	2	41705.54	0.00
Factor4:Factor5:Factor7	72562000	8	32731.62	0.00
Factor1:Factor4:Factor6	56439670	8	25459.08	0.00
Factor5:Factor7:Factor8	55864390	8	25199.58	0.00
Factor4:Factor8	27138830	4	24483.83	0.00
Factor4:Factor7:Factor8	54189780	8	24444.19	0.00
Factor1:Factor5:Factor7	44955540	8	20278.76	0.00
Factor7:Factor8	20447030	4	18446.69	0.00
Factor4:Factor6	20441390	4	18441.61	0.00
Factor4:Factor5:Factor8	40338830	8	18196.23	0.00
Factor1:Factor6	19711770	4	17783.36	0.00
Factor1:Factor4:Factor5	36972850	8	16677.89	0.00
Factor3	8804220	2	15885.8	0.00
Factor4:Factor5	15259490	4	13766.65	0.00
Factor1:Factor5:Factor6	26560900	8	11981.22	0.00
Factor4:Factor5:Factor7:Factor8	51413040	16	11595.82	0.00
Factor5:Factor6:Factor7:Factor8	50624210	16	11417.91	0.00
Factor3:Factor5	11842320	4	10683.78	0.00
Factor5:Factor6:Factor8	23147740	8	10441.59	0.00
Factor4:Factor6:Factor8	22567300	8	10179.77	0.00
Factor4:Factor5:Factor6	22425680	8	10115.89	0.00
Factor1:Factor5:Factor8	21979690	8	9914.708	0.00
Factor4:Factor5:Factor6:Factor7	41881760	16	9446.114	0.00
Factor1:Factor5	10313140	4	9304.199	0.00
Factor1:Factor6:Factor8	20342170	8	9176.046	0.00
Factor1:Factor4:Factor7	20108690	8	9070.726	0.00
Factor1:Factor6:Factor7	18529770	8	8358.497	0.00

Factor6:Factor7:Factor8	15918070	8	7180.399	0.00
Factor1:Factor7:Factor8	13134770	8	5924.896	0.00
Factor4:Factor6:Factor7	12966310	8	5848.905	0.00
Factor1:Factor3	6469976	4	5837.017	0.00
Factor1:Factor4:Factor8	11479170	8	5178.08	0.00
Factor1:Factor4:Factor7:Factor8	20427440	16	4607.255	0.00
Factor1:Factor5:Factor6:Factor7	16346770	16	3686.891	0.00
Factor1:Factor4:Factor6:Factor7	14580700	16	3288.567	0.00
Factor1:Factor4:Factor5:Factor7:Factor8	28287260	32	3189.989	0.00
Factor1:Factor4:Factor5:Factor7	12216420	16	2755.32	0.00
Factor1:Factor4:Factor5:Factor6	12197330	16	2751.016	0.00
Factor6:Factor8	2927707	4	2641.289	0.00
Factor3:Factor4:Factor6	5312120	8	2396.217	0.00
Factor1:Factor4:Factor5:Factor8	10287580	16	2320.285	0.00
Factor1:Factor8	2490460	4	2246.818	0.00
Factor3:Factor4	2487747	4	2244.37	0.00
Factor4:Factor6:Factor7:Factor8	9247359	16	2085.672	0.00
Factor1:Factor3:Factor4	4265709	8	1924.197	0.00
Factor1:Factor5:Factor6:Factor8	8447351	16	1905.236	0.00
Factor4:Factor5:Factor6:Factor8	8192526	16	1847.762	0.00
Factor4:Factor5:Factor6:Factor7:Factor8	14208340	32	1602.292	0.00
Factor3:Factor6:Factor7	3256092	8	1468.774	0.00
Factor1:Factor4:Factor6:Factor8	5928693	16	1337.172	0.00
Factor1:Factor3:Factor8	2931339	8	1322.283	0.00
Factor1:Factor6:Factor7:Factor8	5760538	16	1299.246	0.00
Factor3:Factor8	1326501	4	1196.729	0.00
Factor2	624016.4	2	1125.937	0.00
Factor1:Factor4:Factor5:Factor6:Factor7	9701669	32	1094.069	0.00
Factor1:Factor4:Factor5:Factor6:Factor7:Factor8	18107740	64	1021.016	0.00
Factor1:Factor4:Factor5:Factor6:Factor8	8931969	32	1007.269	0.00
Factor1:Factor4:Factor6:Factor7:Factor8	8719742	32	983.3359	0.00
Factor1:Factor3:Factor4:Factor6	3440146	16	775.8989	0.00
Factor1:Factor5:Factor6:Factor7:Factor8	6591594	32	743.3421	0.00
Factor1:Factor2:Factor6	1566750	8	706.7372	0.00
Factor4	349424.8	2	630.481	0.00
Factor1:Factor5:Factor7:Factor8	2534889	16	571.7251	0.00
Factor3:Factor7:Factor8	1222700	8	551.5415	0.00
Factor2:Factor6	510817.2	4	460.8439	0.00
Factor3:Factor5:Factor6:Factor7	2013062	16	454.0309	0.00
Factor1:Factor3:Factor6:Factor7	1559605	16	351.7571	0.00
Factor3:Factor6:Factor8	645495.2	8	291.1731	0.00
Factor3:Factor4:Factor7	644937.1	8	290.9213	0.00
Factor1:Factor3:Factor6	581136.5	8	262.1419	0.00
Factor1:Factor3:Factor5:Factor7	1143485	16	257.9044	0.00
Factor2:Factor4:Factor7	567743.3	8	256.1004	0.00

Factor2:Factor5:Factor6	508890.2	8	229.5527	0.00
Factor1:Factor2	224143.8	4	202.2157	0.00
Factor3:Factor4:Factor5:Factor6:Factor7	1728961	32	194.977	0.00
Factor3:Factor5:Factor7	403147.2	8	181.8536	0.00
Factor1:Factor3:Factor5	390984.6	8	176.3672	0.00
Factor3:Factor5:Factor6	384334.5	8	173.3675	0.00
Factor3:Factor5:Factor6:Factor7:Factor8	1535168	32	173.1228	0.00
Factor1:Factor3:Factor5:Factor8	755249.5	16	170.3408	0.00
Factor2:Factor8	182077.3	4	164.2646	0.00
Factor1:Factor2:Factor7	350737.5	8	158.2124	0.00
Factor3:Factor4:Factor5:Factor6:Factor8	1362294	32	153.6276	0.00
Factor1:Factor2:Factor6:Factor7	623343.2	16	140.5903	0.00
Factor1:Factor3:Factor5:Factor6:Factor7	1227862	32	138.4675	0.00
Factor1:Factor3:Factor4:Factor7	604475.1	16	136.3348	0.00
Factor3:Factor4:Factor5	280700	8	126.6195	0.00
Factor2:Factor7	136681.6	4	123.31	0.00

For compactness, only terms with nominal $p < 0.05$ are displayed. These nominal p-values are descriptive screening quantities for the ANN-generated surrogate response surface and are not interpreted as evidence of independent experimental statistical significance. **SS: sum of squares; D.f.: degrees of freedom.

Table S6: Results for ANOVA statistical analysis for 100% of CO_2 sequestration capacity data-indirect carbonation*

Factor	SS*	D.f.***	F-value	p-value
Factor5	1595212	2	1448.52	0.00
Factor1:Factor5	1277367	4	579.95	0.00
Factor6	528688.7	2	480.07	0.00
Factor5:Factor6	868696.9	4	394.4056	0.00
Factor2	371015.4	2	336.90	0.00
Factor1:Factor6	485235.2	4	220.31	0.00
Factor1	233933.9	2	212.42	0.00
Factor1:Factor5:Factor6	701937.8	8	159.3468	0.00
Factor4	88424.39	2	80.29	0.00
Factor3:Factor6	172806.9	4	78.45775	0.00
Factor2:Factor6	157583	4	71.54582	0.00
Factor2:Factor5:Factor6	244944.8	8	55.60489	0.00
Factor3:Factor5	113493.1	4	51.5281	0.00
Factor3	56411.35	2	51.22	0.00
Factor2:Factor5	105057.7	4	47.70	0.00
Factor1:Factor2	73492.76	4	33.37	0.00
Factor1:Factor2:Factor5:Factor6	242588.2	16	27.53497	0.00
Factor1:Factor2:Factor5	99777.09	8	22.65039	0.00
Factor1:Factor2:Factor6	99496.9	8	22.58678	0.00
Factor2:Factor3	41613.36	4	18.89	0.00
Factor1:Factor2:Factor3	62341.76	8	14.1522	0.00
Factor1:Factor3:Factor4	59037.83	8	13.40217	0.00
Factor1:Factor2:Factor4	56067.35	8	12.72784	0.00
Factor2:Factor3:Factor4	55585.49	8	12.61846	0.00
Factor3:Factor5:Factor6	53226.23	8	12.08288	0.00
Factor2:Factor3:Factor5:Factor6	105142.3	16	11.93417	0.00
Factor1:Factor2:Factor3:Factor4	100433.3	16	11.39967	0.00
Factor1:Factor3	22859.18	4	10.38	0.00
Factor1:Factor4	21509.57	4	9.77	0.00
Factor1:Factor3:Factor5:Factor6	80723.12	16	9.162475	0.00
Factor3:Factor4	20117.41	4	9.133701	0.00
Factor2:Factor4	16890.01	4	7.67	0.00
Factor2:Factor3:Factor5	29294.11	8	6.650052	0.00
Factor1:Factor2:Factor3:Factor5:Factor6	91885.83	32	5.214749	0.00
Factor1:Factor2:Factor3:Factor5	45910.18	16	5.211033	0.00
Factor2:Factor3:Factor6	18851.47	8	4.279471	0.00

For compactness, only terms with nominal $p < 0.05$ are displayed. These nominal p-values are descriptive screening quantities for the ANN-generated surrogate response surface and are not interpreted as evidence of independent experimental statistical significance. **SS: sum of squares; D.f.: degrees of freedom.

Table S7: Results for ANOVA statistical analysis with 80% of CO_2 sequestration capacity data-indirect carbonation*

Factor	SS*	D.f.***	F-value	p-value
Factor1:Factor5	2675308	4	173407.20	0.00
Factor2	842814.5	2	109258.53	0.00
Factor5	526702.7	2	68279.27	0.00
Factor1	272205.1	2	35287.40	0.00
Factor6	250601.1	2	32486.76	0.00
Factor3:Factor5	413843.1	4	26824.34482	0.00
Factor1:Factor6	235753.3	4	15280.98	0.00
Factor1:Factor5:Factor6	234216.2	8	7590.672252	0.00
Factor2:Factor3:Factor5	212561.1	8	6888.857464	0.00
Factor2:Factor5:Factor6	206820.4	8	6702.807124	0.00
Factor1:Factor2:Factor5	185825.9	8	6022.398981	0.00
Factor2:Factor5	82165.39	4	5325.77	0.00
Factor4	36642.08	2	4750.11	0.00
Factor5:Factor6	71063.01	4	4606.138374	0.00
Factor2:Factor6	67326.77	4	4363.964239	0.00
Factor1:Factor2:Factor5:Factor6	146539.2	16	2374.582896	0.00
Factor1:Factor2	32791.26	4	2125.45	0.00
Factor1:Factor2:Factor3:Factor5	128023	16	2074.53808	0.00
Factor1:Factor2:Factor6	42728.99	8	1384.796755	0.00
Factor2:Factor3	20502.54	4	1328.93	0.00
Factor3	7272.957	2	942.83	0.00
Factor3:Factor6	12478.6	4	808.833438	0.00
Factor4:Factor5	7734.444	4	501.328571	0.00
Factor1:Factor3:Factor5	14904.96	8	483.052411	0.00
Factor3:Factor5:Factor6	12646.56	8	409.860326	0.00
Factor2:Factor3:Factor5:Factor6	22208.77	16	359.880193	0.00
Factor1:Factor3	5479.161	4	355.15	0.00
Factor1:Factor4	2273.323	4	147.35	0.00
Factor1:Factor2:Factor3:Factor5:Factor6	12816.16	32	103.839176	0.00
Factor1:Factor3:Factor5:Factor6	5623.54	16	91.126186	0.00
Factor1:Factor2:Factor3	2136.079	8	69.227816	0.00
Factor2:Factor3:Factor6	2000.821	8	64.844279	0.00
Factor1:Factor4:Factor5	1765.471	8	57.216868	0.00
Factor3:Factor4:Factor5	1463.872	8	47.442381	0.00
Factor1:Factor3:Factor6	1366.18	8	44.276308	0.00
Factor4:Factor5:Factor6	994.8923	8	32.243299	0.00

Factor2:Factor3:Factor4	930.4734	8	30.155555	0.00
Factor1:Factor2:Factor4:Factor5	1726.777	16	27.981418	0.00
Factor1:Factor2:Factor3:Factor6	1455.757	16	23.589699	0.00
Factor2:Factor4	320.1433	4	20.75	0.00
Factor2:Factor4:Factor5	585.2895	8	18.96855	0.00
Factor1:Factor4:Factor5:Factor6	1149.586	16	18.628377	0.00
Factor2:Factor4:Factor5:Factor6	1002.822	16	16.250146	0.00
Factor4:Factor6	220.6787	4	14.303877	0.00
Factor2:Factor3:Factor4:Factor5	715.3552	16	11.591913	0.00
Factor2:Factor3:Factor4:Factor6	469.5239	16	7.60836	0.00
Factor1:Factor4:Factor6	229.4506	8	7.436225	0.00
Factor1:Factor2:Factor4:Factor5:Factor6	792.8989	32	6.424232	0.00
Factor1:Factor2:Factor4:Factor6	257.1819	16	4.167483	0.00
Factor1:Factor3:Factor4:Factor5	246.0311	16	3.98679	0.00
Factor3:Factor4	58.65581	4	3.801933	0.01
Factor1:Factor2:Factor3:Factor4:Factor5	445.9412	32	3.613109	0.00
Factor1:Factor3:Factor4:Factor5:Factor6	348.2664	32	2.821727	0.00
Factor1:Factor3:Factor4	84.48622	8	2.7381	0.01
Factor2:Factor4:Factor6	76.77714	8	2.488257	0.02
Factor3:Factor4:Factor5:Factor6	148.1818	16	2.4012	0.01
Factor1:Factor2:Factor3:Factor4	127.9598	16	2.073514	0.02

For compactness, only terms with nominal $p < 0.05$ are displayed. These nominal p-values are descriptive screening quantities for the ANN-generated surrogate response surface and are not interpreted as evidence of independent experimental statistical significance. **SS: sum of squares; D.f.: degrees of freedom.

Table S8. Final ANN architecture and training controls used for the three process models.

**Parameter**	**Leaching**	**Direct carbonation**	**Indirect carbonation**
Experimental observations	171	168	22
Input variables	7	8	6
Output variable	Extraction efficiency (%)	CO_2 sequestration capacity	CO_2 sequestration capacity
Candidate hidden-layer depths evaluated	10, 20, 50, 100	10, 20, 50, 100	10, 20, 50, 100
Selected hidden layers	100	50	50
Neurons per hidden layer	10	10	10
Final architecture	7-[10 × 100]-1	8-[10 × 50]-1	6-[10 × 50]-1
Hidden-layer activation	tansig	tansig	tansig
Output-layer activation	purelin	purelin	purelin
Trainable parameters	10,981	5,491	5,471
Trainable parameters / observation	≈64	≈33	≈249
Normalization / scaling	mapminmax	mapminmax	mapminmax
Training algorithm	Levenberg–Marquardt	Levenberg–Marquardt	Levenberg–Marquardt
Observation-level split	80/10/10	80/10/10	50/25/25
Model acceptance criterion	Predefined R^2/RMSE thresholds from prior 100-run calibration	Same	Same
Early stopping	10 consecutive validation-error increases	10 consecutive validation-error increases	10 consecutive validation-error increases
Explicit regularization	None documented beyond early stopping	None documented beyond early stopping	None documented beyond early stopping
Reported overall R	0.9816	0.97	0.87
Reported RMSE	≈4.0%	0.03206 g CO_2 g^{-1} slag	0.02826 g CO_2 g^{-1} slag

Note: The original model-development files identify the selected depths and final architectures shown above, but candidate-specific validation/test metrics for the 10/20/50/100 depth sweep were not retained in a reportable form. The parameter-to-observation ratios are included to make model capacity transparent; the indirect-carbonation model is consequently interpreted as exploratory.

Supplementary Note S1. Simplified-ANOVA model specification and residual structure

ANOVA was implemented in Python using statsmodels. An ordinary-least-squares model was fit with statsmodels.formula.api.ols(), with each factor represented categorically [C(Factor1), C(Factor2), …] and interaction terms specified through order (k−1). Type II sums of squares were calculated with sm.stats.anova_lm(model, typ=2). The single highest-order (full k-way) interaction was omitted from the fitted model and pooled into the residual term. For three-level factors, the residual degrees of freedom are therefore $2^7 = 128$ for leaching, $2^8 = 256$ for direct carbonation, and $2^6 = 64$ for indirect carbonation. Because each factorial combination has one deterministic ANN-predicted response and no experimental replication, this residual is not experimental measurement error; it represents variation assigned to the omitted highest-order interaction within the surrogate decomposition. Accordingly, the reported nominal p-values are used only as descriptive screening quantities, whereas normalized F-values are used for relative factor and interaction ranking.
